# Natural hybridization in heliconiine butterflies: the species boundary as a continuum

**DOI:** 10.1186/1471-2148-7-28

**Published:** 2007-02-23

**Authors:** James Mallet, Margarita Beltrán, Walter Neukirchen, Mauricio Linares

**Affiliations:** 1 Galton Laboratory, University College London, Wolfson House, 4 Stephenson Way, London NW1 2HE, UK; 2 Department of Entomology, The Natural History Museum, Cromwell Road, London SW7 5BD, UK; 3 Smithsonian Tropical Research Institute, Balboa, Apartado 2072, Panamá; 4 Winckelmannstrasse 77, 12487 Berlin, Germany; 5 Departamento de Ciencias Biológicas, Instituto de Genética, Universidad de los Andes, Carrera 1E No 18A10, Bogotá, Colombia

## Abstract

**Background:**

To understand speciation and the maintenance of taxa as separate entities, we need information about natural hybridization and gene flow among species.

**Results:**

Interspecific hybrids occur regularly in *Heliconius *and *Eueides *(Lepidoptera: Nymphalidae) in the wild: 26–29% of the species of Heliconiina are involved, depending on species concept employed. Hybridization is, however, rare on a per-individual basis. For one well-studied case of species hybridizing in parapatric contact (*Heliconius erato *and *H. himera*), phenotypically detectable hybrids form around 10% of the population, but for species in sympatry hybrids usually form less than 0.05% of individuals. There is a roughly exponential decline with genetic distance in the numbers of natural hybrids in collections, both between and within species, suggesting a simple "exponential failure law" of compatibility as found in some prokaryotes.

**Conclusion:**

Hybridization between species of *Heliconius *appears to be a natural phenomenon; there is no evidence that it has been enhanced by recent human habitat disturbance. In some well-studied cases, backcrossing occurs in the field and fertile backcrosses have been verified in insectaries, which indicates that introgression is likely, and recent molecular work shows that alleles at some but not all loci are exchanged between pairs of sympatric, hybridizing species. Molecular clock dating suggests that gene exchange may continue for more than 3 million years after speciation. In addition, one species, *H. heurippa*, appears to have formed as a result of hybrid speciation. Introgression may often contribute to adaptive evolution as well as sometimes to speciation itself, via hybrid speciation. Geographic races and species that coexist in sympatry therefore form part of a continuum in terms of hybridization rates or probability of gene flow. This finding concurs with the view that processes leading to speciation are continuous, rather than sudden, and that they are the same as those operating within species, rather than requiring special punctuated effects or complete allopatry. Although not qualitatively distinct from geographic races, nor "real" in terms of phylogenetic species concepts or the biological species concept, hybridizing species of *Heliconius *are stably distinct in sympatry, and remain useful groups for predicting morphological, ecological, behavioural and genetic characteristics.

## Background

### The importance of natural hybridization between species

Recently, major strides have been made in understanding the genetics and ecology of the species boundary in animals. The discreteness, and "reality" of species is being eroded both below and above the level of species. Below the species level, forms are known which remain distinct in spite of potential or actual gene flow. Examples are: host races in phytophagous insects [1,2] and other parasites [3-5], and ecologically or sexually divergent coexisting forms of animals as diverse as sea anemones [6], cicadas [7] fish [8-10], dolphins [11] and killer whales [12]. There is perpetual doubt about the status of related forms which replace one another geographically. New molecular evidence, coupled with revised species concepts has led to taxonomic inflation whereby many readily identifiable taxa, formerly regarded as subspecies within polytypic "biological" species have been upgraded to full species status [13], in spite of abundant hybridization in contact zones. Above the species level, we are beginning to appreciate that hybridization, while rare on a per-individual basis, is a regular and probably important occurrence in nature [14-17]. On average, at least 10% of animal species and maybe 25% of plant species are known to hybridize in nature, although the fraction of species that hybridize may be much higher in rapidly radiating groups [18].

In the past, hybridization was viewed as a secondary phenomenon of little or no evolutionary importance (e.g. ref. [19]: 133). Associated with this view was the idea that actual intermediate stages of speciation could be seen only rarely in nature [20,21], because hybrids were unnatural. This in turn led to a strong emphasis on speciation due to geographic isolation, especially rapid speciation via the "founder effect" [19]. Hybrid zones between differentiated parapatric species or subspecies were therefore interpreted as zones of secondary contact: differentiation was assumed to have occurred in allopatry. Hybridization was even defined by Mayr as "the crossing of individuals belonging to two natural populations that have secondarily come into contact" (ref. [19]: 110). Alternatively, hybrids and hybridization can be viewed as natural intermediate stages of a gradual process of differentiation, possibly in sympatry or parapatry, rather than as unnatural secondary phenomena [14,18,22,23].

Since the evolutionary synthesis, a dominant definition of species in evolutionary biology has been the so-called "biological species concept" [19-21]. Under this concept, members of the same species "actually or potentially interbreed" [19], whereas members of different species cannot do so. Although other, competing definitions of species exist [24,25], most recent studies of speciation claim to have been elaborated and tested using the biological species concept [23,24]. However, "... taxa that remain distinct despite gene exchange have in fact been classified as separate species even by the originators of the biological species concept. Thus there is a clash between two views of species; one is based on the pattern of gene flow, and the other on the maintenance of a cluster of phenotypes ... stable to invasion by foreign genes" [23]. To understand the maintenance of separateness and evolution of species, we need to understand facts about hybridization and gene flow between clusters of phenotypes in nature.

As a part of this movement, many studies have now been done on hybrid zones [23,26,27] and on host races [1,2,28]. However hybrid zone studies have concentrated on parapatric zones of hybridization where hybrids are abundant enough to sample easily. Under the biological species concept, hybridization in such zones is between geographic races, and arguably demonstrates a failure to complete speciation, rather than giving many clues to speciation or species maintenance. In addition, host races can be argued not to be "good species", and therefore could be viewed as having little relevance to interspecific hybridization. Furthermore, even when species that hybridize in sympatry are accepted to be "good species", it could be argued that this is unimportant because no gene flow results; the hybrids may be too sterile or inviable to produce any offspring. Although it is difficult to obtain adequate sample sizes, it would be useful to have more studies of natural hybridization between taxa generally recognized as species, between which natural hybrids are very rare, usually much less than 1%, compared to parental forms from the same area, as well as investigations into back-crossing to parental species.

Here, we review natural interspecific hybridization in a particularly well studied group, neotropical butterflies of the subtribe Heliconiina. Our survey contributes to a reappraisal of the nature of species and speciation. We investigate whether a group of sexual and dioecious animals obey the same fundamental laws of gene flow and introgression as plants and bacteria. Building on a firm base of systematic, genetic, and ecological work on *Heliconius *and their relatives, these data give unrivalled information on the continuum between polymorphisms, races, semi-species, and species in nature.

### Natural hybridization between species of *Heliconius *and *Eueides*

*Heliconius *and related genera are currently classified as subtribe Heliconiina in the Heliconiinae, a subfamily of Nymphalidae [29-32]. Their bright colours and rampant morphological diversification of geographic races within species and between species have led them being highly prized by collectors, and a good representation of specimens is found in museums and private hands worldwide. The Heliconiina are distasteful to predators, and their diverse colour patterns are explained as adaptations for warning colour and Müllerian mimicry. They mimic other butterfly groups, particularly the Ithomiinae, but a substantial fraction mimic unrelated species within the Heliconiina [33,34]. Detailed studies on ecology, behaviour, systematics, mimicry, genetics and speciation of this group have been carried out [29,35-39]. Scattered reports of natural hybrids between *Heliconius *species have appeared [40-47], but this is the first attempt to collate and analyse all known cases of interspecific hybridization across the Heliconiina. We here review hybridization for the whole subtribe, and report many new hybrids, including previously undocumented examples within the genus *Eueides*.

**Figure 1:**
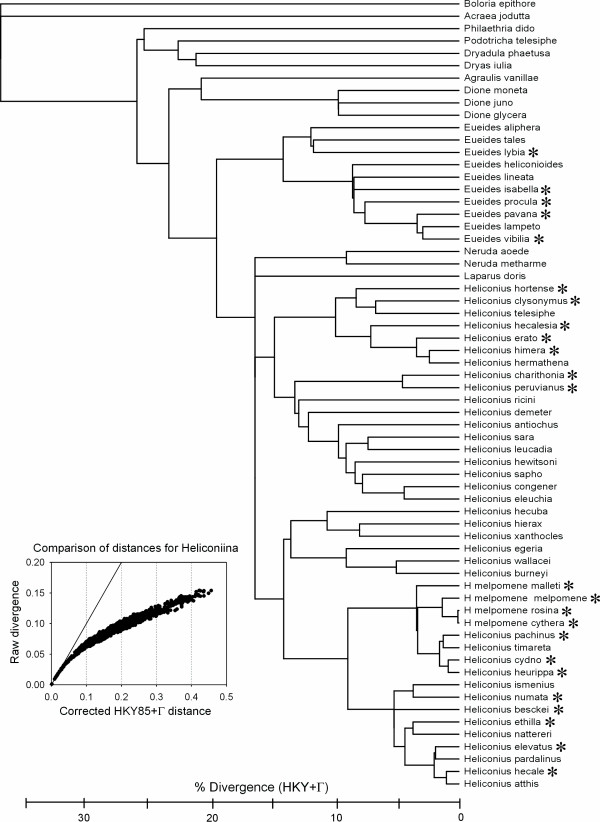
**Phylogenetic relationships in the Heliconiina**. The phylogenetic tree is based on a Bayesian/MCMC consensus tree obtained using a combination of mtDNA (*CoI*+*CoII*, *16S *RNA), and nuclear genes (*elongation factor-1α*, *apterous*, *decapentaplegic *and *wingless*) [30]. * = Species known to hybridize with at least one other species in nature. The tree has been rooted using *Boloria *and *Acraea*. To give an idea of the relative time course of heliconiine evolution, HKY+gamma branch lengths have been estimated using the full likelihood rate-smoothing local molecular clock method of [101] on the *CoI*+*CoII *mitochondrial sequence data alone, after calibrating at the root with the estimated HKY+gamma average divergence between all heliconiines and *Acraea *(0.377).

We put the hybrids into their phylogenetic context. According to morphological [29,32] and molecular evidence [30,31] on the phylogeny of *Heliconius*, the sub-tribe can for our purposes be divided into a number of sub-groups (Fig. 1). There is a basal group of small genera (*Philaethria*, *Agraulis*, *Dione*, *Podotricha*, *Dryadula*, *Dryas*). The genus *Heliconius *and allies form the bulk of the group, consisting of *Eueides *and *Heliconius sensu lato *as sister taxa. *Heliconius sensu lato *consists of four major groups. First there are the "basal species" consisting of two small segregate genera (*Neruda *and *Laparus*) close to *Heliconius sensu stricto*. Some molecular data suggest that these two genera nest within *Heliconius sensu strict*o, but other loci and morphological data suggest they may fall outside *Heliconius *[30,32]; these two lineages are therefore shown provisionally as a polytomy with the two major *sensu stricto *lineages from the base of *Heliconius *in Fig. 1. The third group is the *melpomene-cydno*-silvaniform group, consisting of three probably monophyletic subgroups: (i) the *wallacei/burneyi *and *xanthocles*/*hecuba *subgroup (ii) a "silvaniform" subgroup, in which *atthis*, *hecale*, *ethilla*, *ismenius*, *numata*, and *pardalinus *are mainly Müllerian mimics of the yellow and brown "tiger pattern" Ithomiinae, while *besckei *and *elevatus *have red and yellow more typically heliconiine mimicry patterns; (iii) a *melpomene *subgroup containing *Heliconius melpomene *and *H. cydno*, as well as a handful of segregate "species" – *timareta*, *tristero*, *heurippa*, and *pachinus *– which are probably most closely related to *cydno*. The final, *erato-sara-sapho *group also consists of two parts – the *erato *subgroup and the *sara/sapho/charithonia *subgroup (Fig. 1).

## Results and Discussion

### The data on hybrids between species of *Heliconius*

**Figure 2:**
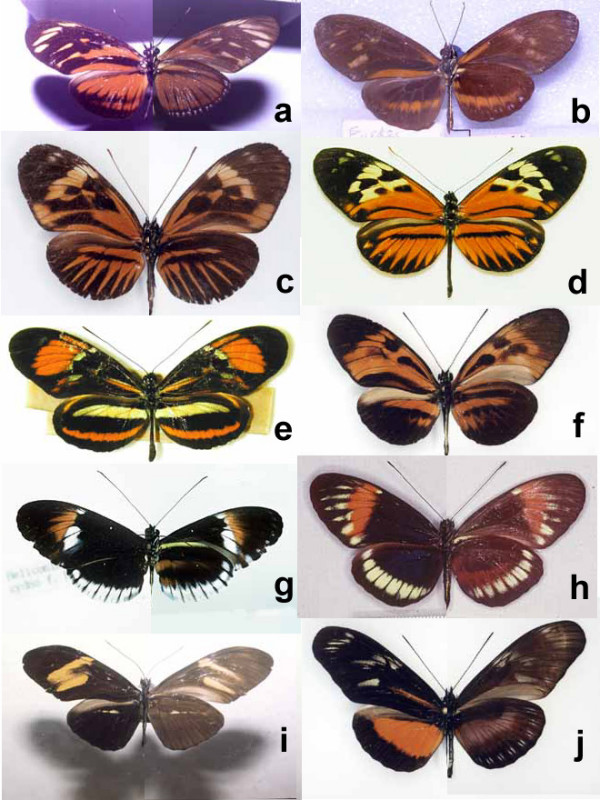
**Newly discovered or little-known interspecific hybrids in *Heliconius *and *Eueides***. **a**. *Eueides isabella eva *× *E. vibilia vialis*, male, hybrid no. 4; **b**. *Eueides isabella eva *× *E. procula vulgiformis*, male, hybrid no. 6; **c**. *Heliconius numata aurora *× *H. melpomene malleti*, female, hybrid no. 11; **d**. *Heliconius hecale zeus *× *H. elevatus perchlorus*, male, hybrid no. 16; **e**. *Heliconius ethilla narcaea *× *H. besckei*, female, hybrid no. 28; **f**. *Heliconius numata superioris *× *H. melpomene meriana*, male, hybrid no. 10; **g**. *Heliconius melpomene cythera *× *H. cydno alithea*, male, hybrid no. 34; **h**. *Heliconius melpomene ssp. nov*. × *H. cydno hermogenes*, female, hybrid no. 65; **i**. *H. erato petiverana *× *H. charithonia vasquezae*, male, hybrid no. 158; **j**. *Heliconius hecalesia octavia *× *H. hortense*, male, hybrid no. 160. For further details, see Table 1 and Additional File 1. All hybrids are putative F_1 _progeny of interspecies hybridization, except *e *which is interpreted as a backcross to *H. besckei*. Photos: *a*, *i *– Sandra Knapp; *b*, *g *– James Mallet; *c*, *f*, *j *– Walter Neukirchen; *d*, *e *– Andrew Brower, *h *– Mauricio Linares.

An extract of the data on hybrid specimens examined is given in Table 1, and images of some previously unpublished or little-known hybrid specimens are shown in Fig. 2. Colour photographs of upper- and undersides of most hybrid specimens on which Table 1 is based are available from Additional File 1. To save space, we display only hybrids; pure forms are illustrated in several useful books which cover the genus [44,48,49]. Detailed lists of known hybrid specimens, discussions of the specimens, laboratory evidence for hybridization, and estimates of frequency in the most abundant forms are given in Additional File 2. Raw mtDNA divergence data [30] are given in Additional File 3. A full database of Additional File 1 is provided as downloadable comma-delimited text in Additional File 4.

**Table 1:** Natural and laboratory hybridization between species of *Heliconius *(see Additional File 1 for specimen details)

Genus	Species 1	Species 2	Geographic relationship	No. of natural hybrids	Backcrossing in lab or field	Laboratory hybrids	Molecular evidence	Assortative mating	F_1 _female sterility
*Eueides*									
	*lybia*	*vibilia*	sympatric	1	-	-	-	(+)	?
	*isabella*	*vibilia*	sympatric	4	-	-	-	(+)	?
	*isabella*	*procula*	sympatric	1	-	-	-	(+)	?
	*pavana*	*vibilia*	sympatric	1	-	-	-	(+)	?
*Heliconius *(*melpomene-cydno*-silvaniform group)									
	*numata*	*melpomene*	sympatric	4	-	-	-	(+)	?
	*ismenius*	*cydno*	sympatric	-	+	+	-	+	+
	*hecale*	*melpomene*	sympatric	2	-	+	-	+	?
	*hecale*	*elevatus*	sympatric	3	+	-	-	(+)	?
	*hecale*	*atthis*	sympatric	-	+	+	-	+	+
	*ethilla*	*melpomene*	sympatric	4	+	-	-	+	?
	*ethilla*	*numata*	sympatric	2	-	-	-	(+)	?
	*ethilla*	*besckei*	sympatric	6	+	-	-	(+)	?
	*melpomene*	*cydno*	sympatric	68	+	+	+	+	+
	*melpomene*	*heurippa*	sympatric	1	+	+	+	+	+
	*melpomene*	*pachinus*	sympatric	-	+	+	+	+	+
	*cydno*	*pachinus*	parapatric	3	+	+	+	+	-
	*cydno*	*heurippa*	parapatric	-	+	+	+	+	-
*Heliconius *(*erato-sara-sapho *group)									
	*himera*	*erato*	parapatric	57	+	+	+	+	-
	*erato*	*charithonia*	sympatric	1	-	-	-	+	?
	*charithonia*	*peruvianus*	parapatric	1	+	-	+	(+)	?
	*hecalesia*	*hortense*	sympatric	1	-	-	-	(+)	?
	*hecalesia*	*clysonymus*	sympatric	1	-	-	-	(+)	?
									
Total				161					

**Figure 3:**
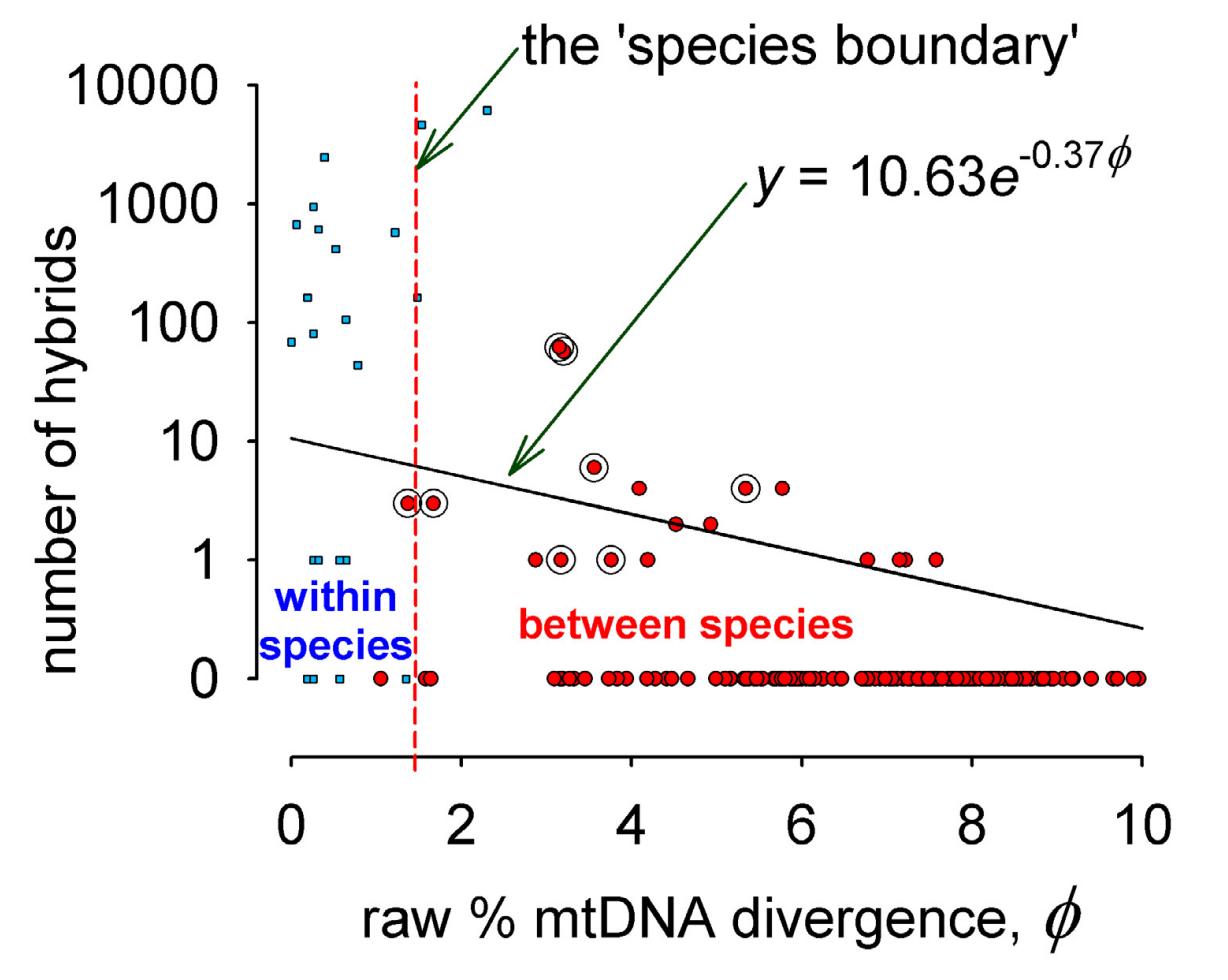
**A graphical representation of the species boundary**. The numbers of natural hybrids known between pairs of species (from Table 1) are plotted on a logarithmic scale against the average uncorrected DNA divergence estimated from data for 1569 bp of mtDNA [30]. If backcrosses are also known from wild specimens, a halo around the point is shown. Comparisons reflect only species that have zones of overlap; average distance measures are given in Additional File 3. There are no known hybrids between species groups, and no estimates of divergence have been included for intergroup comparisons (*Neruda *and *Laparus *are here treated as part of the *melpomene-cydno-*silvaniform group to which they are closest in mtDNA divergence). A least-squares exponential fit of the species data alone is shown. (To display species pairs which lack known hybrids on the log-linear plot, they have been assigned 0.1 hybrids each, but the fitted line is based on a non-linear regression with untransformed data). Because the comparisons are non-independent, especially where branches of the same phylogeny or even the same species are used twice, a simple statistical analysis is not appropriate (under an assumption of independence, there is a highly significant negative correlation between in rates of hybridization and genetic distance: *N *= 180, *P *= 0.0022, although the proportion of the variance explained is not high, *r*^2 ^= 5%, because of the large number of species pairs for which no hybrids are known). Intraspecific hybridization also approximately fits this scheme; smaller square points in blue represent the equivalent numbers of intraspecific hybrids in world collections (not used in curve fitting). These were estimated by counting the numbers of *intraspecific *hybrids (between morphologically divergent subspecies) in the 2001 catalogue of the W. Neukirchen collection, and dividing by the fraction of total *interspecific *hybrids in the Neukirchen collection over the total known worldwide.

Hybrids are unknown from the basal genera of the Heliconiina, or from *Neruda*, *Laparus *and the basal group of *Heliconius*, all of which consist of distantly related species highly divergent from one another at mtDNA (Fig. 1). As many of these species are well known and common, the complete lack of hybrids among basal Heliconiina seems unlikely to be due to sampling bias. All known hybrids belong to the three major recent radiations: *Eueides*, and the *melpomene*-*cydno*-silvaniform and *erato-sara-sapho *groups of *Heliconius*. There is a strong negative correlation between mtDNA divergence and the numbers of hybrids found in the wild (Fig. 3). Backcross hybrids are mainly known in cases of hybridization between the less divergent hybridizing pairs (Fig. 3). Given mtDNA sequence evolution of ~ 2%/My [50], this hybridization and backcrossing suggests the possibility of continued introgression up to 3–4 million years, and sometimes more, after initial divergence (Figs. 1, 3).

### Existence and geographic relations of hybridizing species

It is clear from our data that interspecific hybridization regularly occurs within *Eueides *and *Heliconius*. In a few cases the parents of obvious hybrids are in doubt, for example within *Eueides *or the silvaniforms (Additional Files 1, 2). It is even possible that a few of the more recent hybrids were "manufactured" in captivity for sale to unwary collectors. Yet the majority of specimens we cite here are natural interspecific hybrids of known parentage. We have good evidence for this from many different collectors, and from a large geographic range, including many collections occurring before insectary breeding became widely practised in the 1980s. Although we have uncovered a substantial number of previously unknown hybrids, previous authors have come to similar conclusions about some of the few specimens known previously (Additional File 2). In many cases, we now have laboratory crossing and molecular evidence for hybridization or introgression (Additional File 2)

Most hybrids recorded here are between distinct forms that overlap substantially in their distributions, and are therefore generally considered different species. In three cases, *H. cydno *× *H. pachinus*, *H. erato *× *H. himera*, *H. charithonia *× *H. peruvianus*, the hybridizing taxa are parapatric. We consider these to be species pairs operationally on the grounds that intermediates are rare in areas of contact compared with parental forms (Additional File 2) [37,46,51-55].

The most abundant hybridizations are between very closely related species or sister taxa, for example between *H. melpomene *and *H. cydno*. However, there is plenty of evidence for hybridization between non-sister species, for example between *H. numata *and other silvaniforms and *H. melpomene*. Hybridization of *ismenius*, *hecale, atthis*, *melpomene *and *cydno *in insectaries by Gilbert [47] & Jean-Pierre Vesco (Additional File 1) confirm that such non-sister hybridization is possible and indeed leads to viable backcrossing. Similarly, *H. erato *hybridizes with its sister species *H. himera *wherever the two meet, but also with the more distantly related *H. charithonia *(Fig. 1). The *Eueides *hybrids involving *isabella *and *vibilia *and at least two other species each must also logically involve some non-sister hybridization.

Hybridization and introgression between species is often associated with rapid adaptive radiation on islands; for example in the Darwin's finches on the Galapagos, the Hawaiian silverswords (Compositae) or Hawaiian *Drosophila*, the birds of paradise in New Guinea, cichlids in African lakes, or fish colonists of glacial lakes in the Northern Hemisphere. This study shows that hybridization is not just a feature of island radiations: *Heliconius *is a highly successful genus in the mainland and lowlands of the continent with the most diverse biota on earth. However, hybridizing species are all within *Eueides *and the two major "non-basal" groups of *Heliconius*. These are the three monophyletic groups that appear to be radiating most rapidly compared with less speciose sister groups within the subtribe (Fig. 1); thus, hybridization in *Heliconius *is most likely a general feature of relatively recent radiations, and is not restricted to islands.

### Frequency of hybridization as a fraction of the population

It is clear that the frequency of hybridization is low on a per-individual basis, as in birds: "Hybrids form in only a very minute percentage of the individuals in all the species mentioned, and I know of no case in which the occurrence of hybrids has resulted in a blurring of the border line between these species" ([21]: 262). On the other hand, as Mayr admitted, such statements contain a tautology: "The definition of hybridization as 'the crossing of individuals belonging to two different species' results in circular argument because the decision whether or not to include two populations in the same or in two different species may depend on the occurrence of hybridization" ([19]: 111). Obviously, hybridization and gene flow must be rare whether the biological species concept or even a character-based criterion of species is used, because a total "blurring of the border line" would result in a single species being recognized. Although hybrids must be rare, it is not circular to estimate *how *rare they are. Mayr ([19]: 114) estimated that only one out of 60,000 specimens of birds (across all species) was a true interspecific hybrid. In the birds of paradise, about 30 hybrids were found in 100,000 skins [21], or 0.03%. These values seem about right for *Heliconius *as well. Morphologically detectable hybrids between *H. erato *and *H. himera *form 9.8% of the population in the centre of the best-studied hybrid zone [46], but this is an unusually high rate, and occurs only between two species that replace one another across an extremely restricted hybrid zone. For the most abundantly hybridizing pair of fully sympatric species, *Heliconius melpomene *and *H. cydno*, the fraction of hybrids in natural sympatric populations is usually of the order of 0.05% or less (Additional File 2).

### Frequency of hybridization as a fraction of species

Although hybrid individuals are rare, the frequency of hybridization per species is high. In all, 16 recognized species out of 46 *Heliconius sensu lato *are involved in hybridization, or 35%. (The "*sensu lato*" count includes *Laparus *and *Neruda*, but excludes *Eueides*). Species designations are based on Lamas' checklist [56], except that we here consider *H. hewitsoni *and *H. pachinus *as separate species from *H. sapho *and *H. cydno *respectively. The parapatric *Heliconius cydno*, *H. pachinus, H. heurippa, H. timareta *and *H. tristero *may all be considered to form part of a single species, as could *H. hortense *+ *H. clysonymus*, and *H. sapho *+ *H. hewitsoni*. If these changes are made, hybridization involves 13 species of a total of 40, giving 33% of species hybridizing. For *Eueides*, 5 out of 12 species are involved in hybridization, a fraction of 42%. Overall, there are 73 species of Heliconiina, of which 21 species hybridize, considering *cydno*, and *clysonymus *and *sapho *group species as separate, giving 29%: with the species lumped, 18/67 species hybridize, or 27%. Thus, at least a quarter of all Heliconiina species are involved in natural interspecific hybridization.

The fraction of heliconiine species that hybridize in nature is higher than for animals as a whole (~ 10%), and similar to that of British vascular plants (~ 25%) [18]. However, many smaller groups have higher fractions of hybridizing species than animals as a whole, such as the North American *Papilio *[57], the American warblers, ducks and birds of paradise [18], similar to or exceeding that of the Heliconiina. Even these high rates of hybridization are bound to be underestimates, since there may be many cases in which extremely rare hybrids have remained uncollected, and because hybridization is often hard to detect via morphology in closely similar pairs of species.

### Factors affecting rates of hybridization

It is often said that hybridization between species is distributed patchily among taxonomic groups. According to Mayr ([21]: 260–263, [19]:126–127), natural hybrids in birds are more commonly found in highly dimorphic species such as ducks, game birds, and birds of paradise, that are commonly polygamous or have lekking sexual behaviour. Mayr argued that the short contact period between mates in these species led to more "mistakes". However, this cannot explain high rates of hybridization in the American warblers [18], nor in *Heliconius*, whose males and females mate few times, on average [29,35]. Prager & Wilson [58] used a molecular clock argument to propose that amphibians and birds could remain compatible enough to hybridize for over 20 million years, whereas mammals lose their capacity for hybridization after only 2–3 million years. These authors argue that regulatory gene evolution of intrinsic barriers to hybridization has occurred more rapidly in mammals than in birds or amphibia. However, from broader taxonomic surveys [18], there are few differences between several major taxa in propensity to natural hybridization. The fractions of species known to hybridize in the wild seem not very different among birds, European mammals or European butterflies (9%, 6%, 11%, respectively) [18]. The minor variation among these large surveys is likely to reflect differences in bias or average times of divergence, rather than fundamental differences in regulatory gene action.

A number of biases that affect the per-species estimates of hybridization rate may inflate the apparent heterogeneity. Firstly, colour patterns or other morphology may differ strongly between species in both sexes. This is the case in many brightly coloured birds and butterflies, and it is especially a characteristic of mimetic butterflies such as *Heliconius*. Hybrids will then be more detectable than among drab, relatively uniform taxa. Several other probable examples of such biases have already been given [18]. Thus the apparently high fraction of species hybridizing in ducks, birds of paradise, American warblers, as well as the Heliconiina may not be unusual, or due to the effects of polygamy, but is likely to be closer to the true value because of the greater detectability of hybrids these groups. Sister species will more frequently differ in colour pattern than in drabber groups of comparable size where hybrids would often remain undetected.

On the other hand, it would certainly be surprising if there were no heterogeneity in hybridization among phylogenetic lineages, which could be due to differences in the average ages of sister taxa and speciation rate, as well as inherent effects of the rates of buildup of incompatibilities in different taxa. Within a lineage such as the Heliconiina, the main factor is probably the age of taxa and correlated effects on species compatibility. This seems to be the case here, where no hybrids are known among the older taxa in the "primitive genera" (Figs. 1, 3), and hybridization is restricted to the three most recently diversified groups: genus *Eueides*, the *erato-sara-sapho *group and the *melpomene-cydno-*silvaniform group within the genus *Heliconius*.

### Is hybridization natural?

Mayr [19] argued forcefully that hybridization in the wild was normally due to a "breakdown in isolating mechanisms", particularly after human disturbance of the species' normal habitat. Although this view arises from a somewhat dated view of "isolating mechanisms" as traits beneficial to the species as a whole [59], the argument that hybridization is less intense in pristine habitats is still prevalent today. Clearly, humans might alter habitats in ways which could increase or decrease levels of hybridization. There are frequent conservation concerns when introduced taxa hybridize with native relatives [60,61].

In *Heliconius*, most hybrids are so rare that we cannot for certain say whether they are becoming commoner as a result of habitat alteration. However, many of the hybrid specimens recorded here were collected in the last century or early this century, long before the major episode of rainforest destruction accompanying widespread deployment of the axe and chainsaw. Human activities in rainforests can boost the growth of *Passiflora *foodplants in light gaps, and can greatly change the densities of *Heliconius*, and have probably done so since people arrived in the Americas. However, perhaps one of the clearest examples of human-associated habitat change is the case of a pair of species that probably hybridized more frequently in prehistoric times. The denuded area in Costa Rica now separating *Heliconius cydno *(Atlantic slopes and lowlands) from *H. pachinus *(Pacific slopes and lowlands) should have been suitable for both species. Today the central plateau of Costa Rica lacks suitable rainforest biotopes due to the spread of the capital city of San José in the centre of the probable contact zone [55].

The pair between which we have most hybrid specimens recorded consists of *Heliconius cydno *and *H. melpomene*. Even though the species overlap extensively, *H. cydno *is normally found in small lightgaps or in the understory of lowland tropical forest, and is commoner in uplands to about 1800 m than *melpomene*. *Heliconius melpomene*, on the other hand, is commoner at lower elevations and in more open habitats, such as at the margins of rivers, in savannahs, or scrubby second growth [62,63]. Forest destruction might therefore tend to improve life for *melpomene*, while causing *H. cydno *to retreat. However, while there will have been changes of distribution, and possibly even a temporary increase in contact due to invasion of *melpomene *into habitat with declining populations of *cydno*, there would always have been plenty of contact between the two species in Central America, western Colombia and Ecuador, and in the valleys and slopes of the Andes. An increase in patchy "edge" habitat might have caused hybridization rates between the two species to have changed, but overlap and resultant hybridization almost certainly occurred regularly without human intervention.

The species with the next highest numbers of hybrid specimens, *Heliconius himera *and *H. erato*, are found together only in very narrow zones of overlap. Again, there are habitat differences between the species: *H. himera *is found in higher and drier environments than its close relative *erato *in southern Ecuador and northern Peru [45]. Contacts with hybridization are found in three areas (Additional File 1; [36]). Near Rodríguez de Mendoza in N. Peru, we do not know the exact source of the *H. himera *that hybridizes with the commoner *H. erato *(they probably originate from the Río Marañon drainage near Chachapoyas) so it is unclear whether habitat disturbance has been to blame. In the other two contact zones, in ravines and gallery forests in southern Ecuador and along the Río Marañon in northern Peru, it is likely that contact was more, rather than less extensive before transition zone forests were felled for agriculture. Here, the species are today restricted to steep forested ravines [36,45].

In most other cases of hybridization in the heliconiines there is no obvious reason why hybridization should be solely a result of human interference, even though human-wrought changes in the neotropics have been extensive over the last century. In summary, nothing in the ecology or distribution of any of these species leads one to believe that hybridization started only recently, and only as a result of human habitat disturbance.

### A general law of speciation? The species boundary as an exponential failure law

Is there any evidence for a well-demarcated species boundary in these butterflies? If species have a discrete "reality" of reproductive isolation, we might expect a sharp discontinuity in reproductive isolation between geographic races and species. In Fig. 3, we plot numbers of hybrids known between pairs of races or pairs of species against mtDNA divergence. Rates of hybridization (measured by numbers of hybrids) between species are negatively correlated with the degree of genetic divergence. Assuming that molecular evolution is relatively clock-like, this implies that the frequency of hybridization is related to the time since divergence. This relationship extends even to intraspecific levels. Divergence between members of the same species is usually less than about 1.8% for this region of mtDNA (Fig. 3), and the fitted line therefore predicts even more hybrids between geographic races in collections than between species, as indeed is observed (Fig. 3). In heliconiine butterflies, "reproductive isolation" between populations and between species is not only approximately continuously distributed, but also the effect of genetic divergence between species predicts this relationship.

Although the exact form of the relationship between genetic distance and hybridization probability is not clear from the noisy data available in Fig. 3, the curve is more or less continuous. An increasing failure to hybridize with genetic divergence might be expected to follow an "exponential failure law", the probability distribution that predicts failure of simple mechanical or other devices, such as light-bulbs, with time. An exponential line of fit is plotted in the fitted curve of Fig. 3, and similar log-linear effects of genetic divergence on gene flow occur in transformation experiments with bacteria [64]. An illusion that species are completely reproductively isolated can also be explained by this exponential law: hybrids become too rare to be detected once divergence has proceeded a long way, even though the underlying exponential probability distribution from which hybrids are sampled is actually continuous.

Rather than demonstrating a special effect applying only to eukaryotic, sexual species for which reproductive isolation has some meaning, our data shows that heliconiines approximately follow a log-linear compatibility failure law similar to that found in normally asexual prokaryotes. The chief difference is slope: *Bacillus *exchange genes at a thousandth of the within-strain rate even when chromosomal DNA differs by as much as 20% [64]; in heliconiines, natural hybridization becomes vanishingly rare (i.e. falls bellow the single-hybrid "veil line") beyond about 8% mtDNA divergence (Fig. 3). The difference in slope is not surprising in view of the large differences in biology: failure of bacterial transformation might be due to a lack of uptake of foreign DNA by the bacterial cell wall (although apparently this is not the case in *Bacillus*), or to a failure of the DNA to integrate into the host genome. In heliconiines, failure to produce hybrids depends on behaviour and the probability of mating, and on the fitnesses of hybrid zygotes. Nonetheless, although mechanisms for gene exchange are very different, leading to different slopes, there is an underlying similarity of the species boundary in terms of overall shape and continuity in these very different taxa.

### Evolutionary importance of hybridization

*Heliconius *interspecific hybrid females that have been studied in the laboratory are often sterile, while hybrid males are fertile (Table 1) [47,65,66]. These are examples of Haldane's rule, in which the heterogametic sex (the female in Lepidoptera) suffers greater inviability or sterility than the homogametic sex (the male in Lepidoptera) [67]. The *H. erato *× *H. himera*, *H. pachinus *× *H. cydno *and *H. heurippa *× *H. cydno *hybrids are exceptions that are fertile in both sexes [55,68]. Although female sterility is a characteristic of hybrids between species such as *H. cydno *and *H. melpomene *[47,65,69], Haldane's rule sterility has recently been found between geographic populations considered members of the same species, and even between different populations of the same subspecies (*Heliconius melpomene melpomene *[70]), indicating that even hybrid sterility is not an infallible characteristic of species [71].

In all of the laboratory hybridizations of *Heliconius *studied to date, male hybrids are fertile, even where female hybrids are completely sterile, or sterile in one direction [47,65,66,70]. The presence of backcross hybrids in the wild in a number of these species indicates that introgression may occur, largely in pairs of less divergent species, but even in some rather divergent species. There is clear evidence for natural backcrossing in eight pairs of *Heliconius *species (Fig. 3), representing around 62% of the 13 least divergent hybridizations. In contrast, none of the five most divergent species hybrids show evidence of backcrossing. In the laboratory, backcross broods between *cydno *and *melpomene *and between *erato *and *himera *are fertile, and can be used to introduce genes from one species to another [47,66,72]. Although the initial hybridization can be difficult due to strong assortative mating, genes from *hecale*, *atthis*, *ismenius*, *melpomene *and *cydno *in the *melpomene-cydno-*silvaniform group can apparently be mixed together at will in the laboratory (Additional Files 1, 2; [47]). The similarity of allelic frequencies at some loci, and the strong differences at others in *H. himera *and *H. erato *can be explained by selective gene flow at some loci [51]. Two recent studies [54,55] have demonstrated sharing of some, but not all molecular markers between *Heliconius melpomene*, *Heliconius cydno*, and *H. pachinus*. For example, in both studies, similar or identical haplotypes were found at the autosomal gene *Mpi*, while the same species were entirely distinct at the sex-linked gene *Tpi*. In both studies, also, mitochondrial DNA showed no evidence of introgression, as expected due to Haldane's rule sterility of females. These patterns are best explained by selective introgression at only some genomic regions [54,55].

In *Heliconius*, very similar mimetic colour patterns appear in related, non-sister species, even though closest relatives usually differ in colour pattern [30,33,37]. For example, apparently homologous "ray" mimicry patterns appear in Amazonian *melpomene, timareta*, and *elevatus*, and also the "radiosus" forms of *H. pardalinus *[41]. Possibly, the rayed pattern is ancestral; but, if so, this would require red forewing bands in extra-Amazonian *melpomene*, and in *heurippa*, *tristero*, and *besckei *to have repeatedly evolved in parallel in the different lineages. Multiple parallel evolutionary events may be possible on the *Heliconius *genetic background, but given that DNA introgression occurs, it does not seem unlikely that the occasional hybridization and backcrossing we document has led to transfer of alleles suitable for different mimetic environments. Under this scenario, some of the diversity of mimicry rings achieved by *Heliconius *lineages could be due to their ability to exchange fully formed colour pattern adaptations between closely related species [73,74]; in Gilbert's metaphor, hybridization supplies *Heliconius *species with an interspecific "shared toolkit" of mimicry genes [47]. In addition, because colour pattern is often involved in mate choice [38,39,75], hybridization can lead to new colour pattern combinations which may promote hybrid speciation [76]. This scenario is particularly plausible in *Heliconius heurippa *[66].

An important practical consequence of introgression is that conflicts between morphological or molecular characters in phylogenetic reconstruction may sometimes be explained by gene transfer as well as by parallel evolution and errors in phylogeny estimation. A "true" bifurcating phylogeny of closely related species may not exist, except as an artificial consensus of gene genealogies [54,77,78]. Available multi-locus studies now strongly suggest that introgression selectively affects only certain parts of the genome [54,55,79-82]. In *Heliconius*, the above prediction that horizontal transfer of adaptive colour pattern genes has occurred will become testable when genes affecting colour pattern are characterized at the molecular level [75,83].

### Species continuous with infraspecific forms

Another important lesson from data on hybridization is that species, or at least the entities to which the term "species" is normally applied, may not be completely reproductively isolated, and that speciation does not completely close down gene flow. With time, reproductive barriers will often become more complete, but they may remain leaky in related species, and introgression, even between non-sister taxa, may persist at low levels for many millions of years after speciation. A strict interpretation of the biological species concept might lump all species between which hybrids are known, but this radical solution would require uniting virtually the entire *melpomene-cydno-*silvaniform clade of *Heliconius*, many of the *Geospiza *Darwin's finches, and many species and even genera of ducks, game birds, birds of paradise, orchid genera, among others. Furthermore, if gene flow is our criterion, rather than hybridization, occasional gene flow via horizontal gene transfer is found across even larger systematic divides, especially at the base of the tree of life where it seems to have triggered important adaptive innovations [84]. Yet hybridizing taxa can usually coexist, diversify, radiate and have distinguishable ecologies, sexual behaviour, and genetics, as we expect for species, in spite of this occasional gene flow. Selective gene exchange of this kind now seems likely to be relatively common whenever the weak introgressive pressure expected between species is more than balanced by sufficiently strong disruptive selection (although potentially quite weak in absolute terms) keeping some parts of the genome distinct. This clearly is the case in *Heliconius*: we know of no populations where a pair of hybridizing species form a panmictic hybrid swarm, even though introgression is seen regularly and very likely contributes in important ways to adaptive evolution and speciation.

If the above argument from hybridization against a strict reproductive isolation concept is accepted, it seems clear also that most variants of the phylogenetic species concept will also fail. Given the possibility of gene flow between species taxa, the phylogeny will often be reticulate, even with non-sister taxa, for some while after speciation. A monophyly-based species concept will not do, nor will a concept based on genealogical concordance at multiple loci apply, at least strictly. Instead we are forced to accept that the taxa we name are "unreal" phylogenetic units whose species designations are merely useful because we can tell the clusters of genotypes we call species apart when they overlap, and because they predict distinct morphology, ecology, and behaviour [85]. Such species may have no real species-level phylogeny (except an artificially imposed consensus tree); instead the true reality is that different parts of the genome may have truly different genealogies.

Calling these taxonomic units species might seem unsatisfying to a purist. However, heliconiine species names such as those of the taxa enumerated in Table 1 have been relatively stable since the biosystematic work of Emsley and Brown in the 1960s and 1970s [29,86]. They are also concordant with mate choice, colour pattern, host plant choice, and other ecological parameters known to differ between the species that hybridize [62,63,87]. We have no doubt that, in spite of their leaky boundaries and continuity with infraspecific categories, these heliconiine species taxa and the names applied to them will continue to be useful because the forms they circumscribe are identifiable and because they successfully predict divergent biological traits about which scientists wish to communicate.

## Conclusion

Interspecific hybrids are regularly found among Heliconiina (Lepidoptera: Nymphalidae) in the wild: overall, 26–29% of the species are involved, depending on species concept. Hybrids are restricted to the two most recently radiated "crown" genera, *Heliconius *and *Eueides*, where they involve 33–35% and 42% of species, respectively. These are among the highest recorded per species hybridization rates for any organism [18]. Based on a molecular clock, reproductive isolation can often remain incomplete for more than 3 million years after speciation. Hybridization is, however, rare on a per-individual basis. For one well-studied case of species hybridizing in parapatric contact (*Heliconius erato *and *H. himera*), phenotypically detectable hybrids form around 10% of the population, but for most species in sympatry, hybrids usually form less than 0.05% of individuals. In a few cases for which we have detailed information, backcrossing occurs in the field and fertile backcrosses have been verified in insectaries, which indicates that introgression is likely. Furthermore recent molecular work shows that alleles at some but not all loci are shared between *H. cydno *and *H. melpomene*, a pair of sympatric, hybridizing species [54,55].

Hybridization between species of *Heliconius *appears to be a natural phenomenon; there is no evidence that it has been enhanced by recent human habitat disturbance. There is a roughly exponential decline in the numbers of natural hybrids with genetic distance both between and within species, suggesting an approximation to a simple "exponential failure law" of compatibility as found in some prokaryotes. Geographic races and species that coexist in sympatry therefore form part of a continuum in terms of hybridization rates or probability of gene flow. Although not qualitatively distinct from geographic races, nor "real" in terms of phylogeny or lack of hybridization, species must by definition be identifiable via some loci that are stably divergent in sympatry. Named heliconiine species remain useful for predicting significant differences in morphological, ecological, behavioural and genetic characteristics, even though they regularly hybridize and exchange genes. This finding concurs with the view that processes leading to speciation are continuous, rather than sudden, and that they are the same as those operating within species, rather than requiring special punctuated effects or complete allopatry. Furthermore, the transfer of adaptive genes is possible, and may play an important role in adaptation and speciation.

## Methods

### Detection and definition of "hybrids"

Data and photographs of specimens noted here from literature records, museums, and private collections were collated into a database (Additional Files 1, 4). A few other interspecific *Heliconius *hybrids may still exist in smaller public and private collections not visited by us. However, we believe our extensive international coverage is adequate for the purpose of documenting the extent of hybridization across the genus.

Closely related species of *Heliconius *usually belong to distinct mimicry rings [30,33,37], suggesting that a shift in mimicry plays a role in speciation and the maintenance of specific distinctness thereafter [35,37,38]. Therefore, putative hybrids between such species are mostly easy to identify. Having located potential hybrid specimens, we use morphological criteria, coupled with knowledge of intra- and interspecific *Heliconius *genetics [72,83,88-93] to decide whether they constitute hybrids or intraspecific variants. This is not always easy. Hybridization or introgression between species can cause difficulties in defining the species themselves, let alone their hybrids and intergrades. We here define the term "hybrids" and "pure species" operationally via morphology, molecular genetic data where available, and knowledge of the colour pattern genetics: "pure species" are usually known from hundreds of individuals, and, in heliconiines, their biology will usually be documented. Even if rare, a pure species is often numerous in some areas, and only rarely is polymorphic within any one area (exceptions to this rule exist: for example *H. numata *and *H. cydno *exhibit local mimetic polymorphisms [94-97]). "Hybrids" are highly unusual phenotypes from well outside the normal range of variation of known species that are most easily interpreted as progeny of crosses between two known species because of a combination of traits from each. First generation (F_1_) hybrids are readily distinguished providing the parent species are sufficiently distinct in morphology. Colour pattern differences often depend on relatively few loci: this is the case for the geographic forms of *Heliconius melpomene*, *H. erato*, and *H. numata *[83,88,89,98] as well as between *H. erato *and *H. himera *[37,45,90,92] and between *H. melpomene *and *H. cydno *[47,72,98]. If there are backcrosses, they and F_2 _progeny can potentially recreate the full range from parental phenotypes to F_1_-like "obvious" hybrids [72]. Therefore, we use the designation "F_1_" to mean that the phenotype could have been produced as a first generation cross (though it may sometimes actually have been produced by a backcross or F_2_), and by "backcross" we mean all other hybrids that do not have the F_1 _phenotype [46]. Since hybridization is usually very rare for any pair of species, it is likely that almost all "F_1_s" are actually first generation hybrids, and most "backcrosses" are offspring of actual F_1_s backcrossed to a parental species (although some backcrosses will be missed among "F_1_s" and among "pure" specimens).

A number of interspecific hybridizations have now been studied in insectaries, giving evidence useful both for establishing the likelihood of hybridization, and to demonstrate the potential range of phenotypes. The detailed series of crosses between *H. erato *and *H. himera*, and between *H. cydno *and *H. melpomene *have been mentioned above. Several other hybridizations have also occurred, more or less accidentally, in insectaries. A major series involves *Heliconius ismenius, H. hecale, H. melpomene*, *H. cydno *and *H. pachinus *[37,47,99]. In another example, Jean-Pierre Vesco (pers. comm.) obtained hybrids between a male *H. hecale *from Costa Rica and a female *H. atthis *from W. Ecuador. The F_1 _hybrid males were successfully backcrossed to females of both parents: *H. atthis*, *H. hecale*, and also outcrossed to a third species, *H. melpomene *(which itself had some colour pattern elements obtained by hybridization with *H. cydno*). Vesco reports that many of the female hybrids were sterile, so the crosses were obtained only via backcrosses with male hybrids (Vesco's photos are obtainable via Additional File 1).

Speciation requires genetic divergence, but there is always the possibility that alleles now common in one species remain as low frequency ancestral polymorphisms in a sister species. It is therefore hard to differentiate rare ancestral polymorphism, potentially augmented by mutation, from polymorphisms introduced by introgression (i.e. hybridization and back-crossing). We use two major criteria to decide whether a specimen is a hybrid. First, specimens showing two or more presumably independent hybrid characteristics strongly implicate hybridization as a cause. If rare ancestral alleles or mutation-derived phenocopies of genetic traits in another species are present in the absence of hybridization, it is very unlikely that two or more such traits will be found in the same individual provided that genetic loci coding for the variation are independent; for example, if each putatively hybrid trait has frequency 0.1% (a generous estimate for the frequency of the commonest hybrid phenotypes, for example the frequency of red forewing bands putatively from *Heliconius melpomene *within *Heliconius cydno *– see Results and Discussion), two such traits should be found at a frequency of one in a million, and three traits at a frequency of only one in a billion. In true hybrids, on the other hand, hybrid traits will normally be found together. In the cases were hybrids are reasonably common and easy to produce (within the *Heliconius erato *or *H. melpomene *groups, for example), analysis of genetic architecture confirming such independence of characteristics has been carried out in the laboratory, and in some cases molecular studies have also confirmed the existence of introgression [46,54,55,72,73,88,90]. Normally, we identified hybrids by means of comparisons of their external phenotype, using laboratory crosses as a guide where these are available, but in some of the commonest cases of interspecific hybridization we have molecular genetic evidence of hybrids, as detailed in Table 1 and Additional File 2.

As well as the correlation of hybrid phenotypes within individuals, we also use correlations between the location of capture of hybrids and the geographic distributions of putative parental species and races as supporting evidence for hybrid status. For instance, the existence of red *melpomene*-like forewing bands in specimens otherwise similar to *H. cydno *might be due to ancestral polymorphism, because the two species are sister taxa (Fig. 1). However, a putative hybrid between *Heliconius cydno *and *H. melpomene *would be highly unlikely in Brazil or the Cauca Valley of Colombia because only *melpomene *is present in Brazil, and only *cydno *in the Cauca Valley. If on the other hand, potential hybrid variants are due to hybridization, such phenotypes should be present only in extra-Amazonian areas where both *H. cydno *and *H. melpomene *are present and where the latter has a red forewing band (as in fact they are). This geographic aid to hybrid identification is further enhanced because the species acting as parents of hybrids consist of as many as 30 very strongly divergent geographic races distinguished by colour pattern.

### The potential for fraudulent hybrids manufactured in captivity

A possible consequence of the interest that these rare natural hybrids now generate on the international butterfly market is that there is a financial incentive to offer captive-bred hybrid specimens for sale with fraudulent locality labels. Bred hybrids seem most likely from the late 1980s onwards, when "butterfly houses" and commercial breeding facilities in the tropics supplying livestock became more widespread. The specimens tabulated and figured here were largely collected before this time. We can be certain that the older specimens are genuine, since multiple-generation *Heliconius *culture was unknown before the 1950s, and practised only by a handful of academic *Heliconius *biologists before the 1980s. Post-1980s specimens could be more dubious, and we have used only specimens whose provenances seem impeccable; we have visited key sites in Colombia, Costa Rica, and Panama, and have personally communicated with some of their collectors (León Denhez, Diego Torres, and Rodrigo Torres in the Cali area, Ernesto Schmidt-Mumm and Jean François LeCrom in Bogotá, José Urbina in Otanche, and Adolfo Ibarra in México).

### Mitochondrial DNA divergence

DNA sequence information has been obtained for almost all species of Heliconiina [30,31,100]. In this paper we use data from 1569 bp of mitochondrial DNA of the genes *CoI*, *tRNA-leu*, and *CoII *[30] to estimate genetic divergences. Mitochondrial sequences in Lepidoptera are a particularly useful standard for genetic divergence both within and between species, for two reasons. Firstly, there is thought to be no recombination between mitochondria, due to unisexual inheritance; thus genetic divergence is unlikely to be affected by occasional introgression. Secondly, in *Heliconius*, as in many Lepidoptera [67], hybrid females are often sterile, an example of Haldane's rule. Haldane's rule will ensure that introgression of maternally inherited mitochondria is prevented at an earlier stage of speciation than for nuclear loci [38,57,65,70]; the latter may transferred between species by backcrossing of male hybrids.

## Authors' contributions

James Mallet conceived the idea, collated and analysed most of the data, and wrote the majority of the text. Margarita Beltrán performed the majority of the molecular work for the phylogenetic analysis of Fig. 1. Walter Neukirchen and Mauricio Linares helped to collate major groups of hybrid specimens from world Museums, and also from Linares' and especially Neukirchen's own collections, and made substantial contributions to writing the paper.

## Supplementary Material

Additional File 1 **Hybrids between species of *Heliconius *and *Eueides *butterflies: a database**. HTML file linking to database of all known wild-caught interspecific hybrid specimens in the *Heliconiina*, consisting of introductory text, a list of specimens, together with collection data and photographs of the specimens, and links to information about some artificial hybrids and mutants in the group. This is an edited copy of our online database of *Heliconius *hybrids [102]. To view database, download zip file and extract to a separate folder, then open index.html within that folder. Click here for file

Additional File 2 **Discussion of individual hybrid specimens**. PDF document giving detailed evidence for the specimens in the hybrid database. Click here for file

Additional File 3 **Distance measures for mtDNA among species of Heliconiina**. PDF document containing table with details of average raw % DNA divergence, based on 1569 bp of the genes *CoI*, *tRNA-leu*, and *CoII*. Click here for file

Additional File 4 **Complete data for hybrids shown in **Additional File 1. CSV (comma-delimited text) document with complete data recorded for each hybrid specimen. This is the same as the data for Additional File 1, except that the data for web presentation in that HTML version is abbreviated for ease of presentation. Click here for file
