# Supplementary material for: Natural hybridization in heliconiine butterflies: the species boundary as a continuum
Source: BMC Evol Biol. 2007 Feb 23;7:28. doi: 10.1186/1471-2148-7-28 (PMC1821009; doi:10.1186/1471-2148-7-28)
Supplement: Additional File 1 — Hybrids between species of Heliconius and Eueides butterflies: a database. HTML file linking to database of all known wild-caught interspecific hybrid specimens in the Heliconiina, consisting of introductory text, a list of specimens, together with collection data and photographs of the specimens, and links to information about some artificial hybrids and mutants in the group. This is an edited copy of our online database of Heliconius hybrids [102]. To view database, download zip file and extract to a separate folder, then open index.html within that folder. [file 1471-2148-7-28-S1.zip › artif/artifhyb.html]

Artificial hybrids between Heliconius species


# Artificially produced hybrids between species and races of *Heliconius* butterflies


## James Mallet

Back to: "**Hybridization
and the nature of species ...**"
  
**Database of
natural *Heliconius* hybrids**
  


---

**Personal communications about *Heliconius*
hybrids produced in captivity**

During our compilation of the database of natural
interspecific hybrids in collections, we have excluded any hybrids certainly or
probably produced in captivity.  (We have also excluded any intraspecific
hybrids. There are now a number of butterfly farms and butterfly houses in which
multiple species of *Heliconius* from different regions are kept together
at high density, and hybrids can often result in these situations.  In addition,
there is now a certain amount of enthusiasm among purchasers of exotic Lepidoptera
for varieties and hybrids, and it therefore seems not improbable that some of
the commercial market in such rarities will be filled by artificial hybrids. 
*Caveat emptor*!

Luis Miguel Constantino
[*H.heurippa, H.cydno, H. melpomene, H. charitonia*].   
Jean-Pierre Vesco [*H.melpomene,
H.cydno, H.hecale, H.atthis*].  
Lawrence E. Gilbert [*H. melpomene* x *H.
cydno* gynandromorph]

---

Back to: "Hybridization and
the nature of species ..."   
Source: J.
Mallet

**Last updated:** 18 September 2006
  
 
  
 

  
  
