# Supplementary material for: Natural hybridization in heliconiine butterflies: the species boundary as a continuum
Source: BMC Evol Biol. 2007 Feb 23;7:28. doi: 10.1186/1471-2148-7-28 (PMC1821009; doi:10.1186/1471-2148-7-28)
Supplement: Additional File 1 — Hybrids between species of Heliconius and Eueides butterflies: a database. HTML file linking to database of all known wild-caught interspecific hybrid specimens in the Heliconiina, consisting of introductory text, a list of specimens, together with collection data and photographs of the specimens, and links to information about some artificial hybrids and mutants in the group. This is an edited copy of our online database of Heliconius hybrids [102]. To view database, download zip file and extract to a separate folder, then open index.html within that folder. [file 1471-2148-7-28-S1.zip › artif/ch01.html]

artificial cydno interracial hybrid 1


---


  
Interracial hybrid between *Heliconius
cydno cydnides* and *Heliconius cydno weymeri*
  
Colombia
  
© Luis M. Constantino 2001


Return to: Index
of L.M. Constantino's *Heliconius* hybrids
  
Return to: Other
artificial *Heliconius* hybrids

To
next hybrid
  
To
previous hybrid

**Last updated:**
15 May 2001 


---
