# Supplementary material for: Natural hybridization in heliconiine butterflies: the species boundary as a continuum
Source: BMC Evol Biol. 2007 Feb 23;7:28. doi: 10.1186/1471-2148-7-28 (PMC1821009; doi:10.1186/1471-2148-7-28)
Supplement: Additional File 1 — Hybrids between species of Heliconius and Eueides butterflies: a database. HTML file linking to database of all known wild-caught interspecific hybrid specimens in the Heliconiina, consisting of introductory text, a list of specimens, together with collection data and photographs of the specimens, and links to information about some artificial hybrids and mutants in the group. This is an edited copy of our online database of Heliconius hybrids [102]. To view database, download zip file and extract to a separate folder, then open index.html within that folder. [file 1471-2148-7-28-S1.zip › artif/constantino.html]

Luis Miguel Constantino's Heliconius hybrids

**Luis
Miguel Constantino's *Heliconius* hybrids**
  


---

The following were raised by L.M. Constantino
in his insectaries or, in the case of interracial *Heliconius* *cydno*
hybrids, collected in nature. I am grateful to Mr. Constantino for information
and permission to use these photographs.

(Click on specimens below to go to higher
resolution pictures and explanations)


|  |  |  |  |  |  |
| --- | --- | --- | --- | --- | --- |
|  |  |  |  |  |  |
|  |  |  |  |  |

 (© Luis M. Constantino
2001)


---

Back to: Artificial
hybrids ...
  
Source: J.
Mallet
