# Supplementary material for: Natural hybridization in heliconiine butterflies: the species boundary as a continuum
Source: BMC Evol Biol. 2007 Feb 23;7:28. doi: 10.1186/1471-2148-7-28 (PMC1821009; doi:10.1186/1471-2148-7-28)
Supplement: Additional File 1 — Hybrids between species of Heliconius and Eueides butterflies: a database. HTML file linking to database of all known wild-caught interspecific hybrid specimens in the Heliconiina, consisting of introductory text, a list of specimens, together with collection data and photographs of the specimens, and links to information about some artificial hybrids and mutants in the group. This is an edited copy of our online database of Heliconius hybrids [102]. To view database, download zip file and extract to a separate folder, then open index.html within that folder. [file 1471-2148-7-28-S1.zip › artif/constantino1.html]

Luis Miguel Constantino's Heliconius hybrids

**Luis
Miguel Constantino's *Heliconius* hybrids**
  


---

  
  
(© LM Constantino 1999)

I am grateful to Luis Miguel Constantino
for sending me these pictures of hybrids in his collection.  In spite
of the label, these hybrids appear to involve various races of *H. cydno*
from W. Colombia, Cauca and Magdalena Valleys, and *H. heurippa* from
near Villavicencio.  The form of the forewing red suggests *H. heurippa*
rather than *H. melpomene* as the likely parent.
  


---

  
Return to: Index
of L.M. Constantino's *Heliconius* hybrids
  
Return to: Other
artificial *Heliconius* hybrids
