# Supplementary material for: Natural hybridization in heliconiine butterflies: the species boundary as a continuum
Source: BMC Evol Biol. 2007 Feb 23;7:28. doi: 10.1186/1471-2148-7-28 (PMC1821009; doi:10.1186/1471-2148-7-28)
Supplement: Additional File 1 — Hybrids between species of Heliconius and Eueides butterflies: a database. HTML file linking to database of all known wild-caught interspecific hybrid specimens in the Heliconiina, consisting of introductory text, a list of specimens, together with collection data and photographs of the specimens, and links to information about some artificial hybrids and mutants in the group. This is an edited copy of our online database of Heliconius hybrids [102]. To view database, download zip file and extract to a separate folder, then open index.html within that folder. [file 1471-2148-7-28-S1.zip › artif/edias_not.html]

hybrid not edias


---


  
?Hybrid or ?mutant form of *Eueides* sp.
  
This female specimen is in the Natural History Museum, London,
  
and is labelled "New Granada, Hewitson coll., *Eueides edias*".
  
A note from Keith S. Brown nearby says it isn't *edias*, but also that he doesn't know what it is.

Return to "Hybrids .." document  
Go to: Mutant
heliconiines

**Last updated:** 10 October 2002

---

  
