# Supplementary material for: Natural hybridization in heliconiine butterflies: the species boundary as a continuum
Source: BMC Evol Biol. 2007 Feb 23;7:28. doi: 10.1186/1471-2148-7-28 (PMC1821009; doi:10.1186/1471-2148-7-28)
Supplement: Additional File 1 — Hybrids between species of Heliconius and Eueides butterflies: a database. HTML file linking to database of all known wild-caught interspecific hybrid specimens in the Heliconiina, consisting of introductory text, a list of specimens, together with collection data and photographs of the specimens, and links to information about some artificial hybrids and mutants in the group. This is an edited copy of our online database of Heliconius hybrids [102]. To view database, download zip file and extract to a separate folder, then open index.html within that folder. [file 1471-2148-7-28-S1.zip › artif/jpvesco.html]

Jean-Pierre Vesco's Heliconius hybrids

**Jean-Pierre
Vesco's *Heliconius* hybrids**
  


---

These specimens were all reared  by Jean-Pierre
Vesco in his insectaries.  I am grateful to Mr. Vesco for information
and permission to use these photographs.

(Click on specimens below to go to higher
resolution pictures and explanations)


|  |  |  |  |  |  |
| --- | --- | --- | --- | --- | --- |
|  |  |  |  |  |  |
|  |  |  |  |  |

(© Jean-Pierre Vesco 2001)

The upper two photos on the left show *Heliconius
hecale* [Costa Rica]x
*atthis*
F1 hybrids. The third upper photo shows *Heliconius* (*hecale*
[Costa Rica]x
*atthis*)
backcrossed to *atthis*.The
three upper pictures on the right show *Heliconius* (*hecale*
[Costa Rica]x
*atthis*)
backcrossed to *hecale* [Costa Rica].The
lower pictures show *Heliconius* (*hecale* [Costa Rica]x
*atthis*)
x *melpomene* [the
variable *melpomene* stock had acquired some *cydno* white forewing
band genes after hybridization about 10 generations previously].
  


---

  
Back to: Artificial
hybrids ...
  
Source: Jim
Mallet Home Page
