# Supplementary material for: Natural hybridization in heliconiine butterflies: the species boundary as a continuum
Source: BMC Evol Biol. 2007 Feb 23;7:28. doi: 10.1186/1471-2148-7-28 (PMC1821009; doi:10.1186/1471-2148-7-28)
Supplement: Additional File 1 — Hybrids between species of Heliconius and Eueides butterflies: a database. HTML file linking to database of all known wild-caught interspecific hybrid specimens in the Heliconiina, consisting of introductory text, a list of specimens, together with collection data and photographs of the specimens, and links to information about some artificial hybrids and mutants in the group. This is an edited copy of our online database of Heliconius hybrids [102]. To view database, download zip file and extract to a separate folder, then open index.html within that folder. [file 1471-2148-7-28-S1.zip › artif/jpvesco1.html]

Jean-Pierre Vesco's Heliconius hybrids

**Jean-Pierre
Vesco's *Heliconius* hybrids**
  


---

From: JPVesco@...
  
Date: Fri, 23 Mar 2001 16:24:53 EST
  
Subject: Re: *Heliconius* hybrids
  
To: J.Mallet@...

Dear James,

I am very interested by your project and
web-site, and I shall try to help you.

About *Heliconius* hybridisation,
all were obtained in a little greenhouse (100sq meters), and were spontaneous
: no tentative of artificial hand-pairing.

I have a very polymorphic *melpomene*
population captive bred for 10 years, resulting from various crosses between
different races (French Guiana, Costa Rica, Ecuador).

A few years ago, I introduced a new species
from Costa Rica : *H. cydno*. Only one female flew correctly and immediately
a male *melpomene* had paired it, and I could obtain 5 hybrids (3
different patterns due to the great genetic variability of *melpomene*,
I obtained a population of *melpomene* with white spots (colour unknown
in *melpomene*).

Last year I obtained *hecale* X *atthis*
hybrids and back-cross with *hecale*, and more surprisingly a fertile
pairing of this hybrid whith a female *melpomene*. Of course the children
are very curious, and sucessfully paired with *melpomene*.

I have taken some pictures and can duplicate
them if you are interested.

Best wishes
  
J Pierre Vesco
  


---

  
From: JPVesco@...
  
Date: Fri, 30 Mar 2001 02:10:00 EST
  
Subject: Re: Heliconius hybrids
  
To: J.Mallet@...

Dear James

... regarding the hybrid[ization] with
*cydno*,
when I speak about the white spot, it is only for the large spot on the
anterior wing, and not on the posterior, because the race used, is from
Costa rica whith only one big spot and nothing on the posterior.

The *hecale* used are from Costa Rica
(*zuleika*).

sincerely yours
  
jp vesco
  


---

  
Back to: "Artificial hybrids
..."   
Source: Jim
Mallet Home Page
