# Supplementary material for: Natural hybridization in heliconiine butterflies: the species boundary as a continuum
Source: BMC Evol Biol. 2007 Feb 23;7:28. doi: 10.1186/1471-2148-7-28 (PMC1821009; doi:10.1186/1471-2148-7-28)
Supplement: Additional File 1 — Hybrids between species of Heliconius and Eueides butterflies: a database. HTML file linking to database of all known wild-caught interspecific hybrid specimens in the Heliconiina, consisting of introductory text, a list of specimens, together with collection data and photographs of the specimens, and links to information about some artificial hybrids and mutants in the group. This is an edited copy of our online database of Heliconius hybrids [102]. To view database, download zip file and extract to a separate folder, then open index.html within that folder. [file 1471-2148-7-28-S1.zip › chaper01.html]

hybrid chaper01


---


Hybrid between *Heliconius charithonia charithonia* and*H. peruviana*
Ecuador
© James Mallet

Return to table
of hybrids

To next hybrid
  
To previous hybrid

```
NOTES

No:                      159
Genus of species 1:      Heliconius
Species 1:               charithonia
Subspecies of species 1: charithonia
Genus of species 2:      Heliconius
Species 2:               peruvianus
Subspecies of species 2:
Sex:                     m
Country:                 Ecuador
Locality:                Pichincha: R�o Toachi, 1700m
Year:                    1996
Photo no.:               chaper01
Named hybrid:
Collection:              Jiggins
Collector:               C.Jiggins
Author/publication:      Jiggins & Davies 1998
Notes:                   BC->charithonia (allozymes); sparse overlap/hybridization, on basis of allozyme data
```

**Last updated:** 18 October 2003

---
