# Supplementary material for: Natural hybridization in heliconiine butterflies: the species boundary as a continuum
Source: BMC Evol Biol. 2007 Feb 23;7:28. doi: 10.1186/1471-2148-7-28 (PMC1821009; doi:10.1186/1471-2148-7-28)
Supplement: Additional File 1 — Hybrids between species of Heliconius and Eueides butterflies: a database. HTML file linking to database of all known wild-caught interspecific hybrid specimens in the Heliconiina, consisting of introductory text, a list of specimens, together with collection data and photographs of the specimens, and links to information about some artificial hybrids and mutants in the group. This is an edited copy of our online database of Heliconius hybrids [102]. To view database, download zip file and extract to a separate folder, then open index.html within that folder. [file 1471-2148-7-28-S1.zip › cydpac03.html]

hybrid cydpac03


---


  
Hybrid between *Heliconius cydno galanthus* and *H. pachinus*
  
Costa Rica
  
© M. Kronforst 

Return to table of hybrids

To next hybrid
  
To previous hybrid

```
NOTES

No:                      100
Genus of species 1:      Heliconius
Species 1:               cydno
Subspecies of species 1: galanthus
Genus of species 2:      Heliconius
Species 2:               pachinus
Subspecies of species 2:
Sex:                     m
Country:                 Costa Rica
Locality:                Heredia: La Selva Biological Station
Year:                    17 July 2002
Photo no.:               cydpac03
Named hybrid:
Collection:              M. Kronforst
Collector:               K. Kronforst
Author/publication:
Notes:                   BC->cydno
```

**Last updated:** 14 June 2006

---
