# Supplementary material for: Natural hybridization in heliconiine butterflies: the species boundary as a continuum
Source: BMC Evol Biol. 2007 Feb 23;7:28. doi: 10.1186/1471-2148-7-28 (PMC1821009; doi:10.1186/1471-2148-7-28)
Supplement: Additional File 1 — Hybrids between species of Heliconius and Eueides butterflies: a database. HTML file linking to database of all known wild-caught interspecific hybrid specimens in the Heliconiina, consisting of introductory text, a list of specimens, together with collection data and photographs of the specimens, and links to information about some artificial hybrids and mutants in the group. This is an edited copy of our online database of Heliconius hybrids [102]. To view database, download zip file and extract to a separate folder, then open index.html within that folder. [file 1471-2148-7-28-S1.zip › erahim55.html]

hybrid erahim55


---


Hybrid between *Heliconius erato cyrbia* and *H. himera*
Ecuador
© P. Boyer

Return to table
of hybrids

To next hybrid
  
To previous hybrid

```
NOTES

No:                      150
Genus of species 1:      Heliconius
Species 1:               himera
Subspecies of species 1:
Genus of species 2:      Heliconius
Species 2:               erato
Subspecies of species 2: cyrbia
Sex:                     m
Country:                 Ecuador
Locality:                El Oro: Guayquichuma
Year:                    2001
Photo no.:               erahim55
Named hybrid:
Collection:              Boyer
Collector:               P. Boyer
Author/publication:
Notes:                   F1
```

**Last updated:** 18 October 2003

---
