# Supplementary material for: Natural hybridization in heliconiine butterflies: the species boundary as a continuum
Source: BMC Evol Biol. 2007 Feb 23;7:28. doi: 10.1186/1471-2148-7-28 (PMC1821009; doi:10.1186/1471-2148-7-28)
Supplement: Additional File 2 — Discussion of individual hybrid specimens. PDF document giving detailed evidence for the specimens in the hybrid database. [file 1471-2148-7-28-S2.pdf]

Natural hybridization in heliconiine butterflies: the species boundary as a continuum

by James Mallet, Margarita Beltrán, Walter Neukirchen, and Mauricio Linares

Additional file 2 – Discussion of individual hybrid specimens

Note: "Hybrid nos." given in the text refer to the ID of individual hybrids in the database of Additional Files 1 & 4.

Hybrids in *Eueides* (hybrid nos. 1-7)

We know of only a few hybrids within *Eueides*, and none have been hitherto recorded in the scientific literature. No laboratory crosses have been carried out, so the correct identification of parents of these *Eueides* hybrids is less certain than those in the genus *Heliconius*. Firstly, hybrid no. 1 from Brazil is clearly similar to *E. lybia* from the underside patterning, although many typical markings such as red spots are obscured, as expected in a hybrid. The other parent of hybrid no. 1 is more obscure; the very dark forewing distal region suggests that the abundant Amazonian form *E. vibilia unifasciatus* as the other parent. Hybrid nos. 2-5 between *E. isabella* and *E. vibilia* were detected as hybrids by Mexican collectors, and are known only from four specimens from Central and Southern Mexico in the collection of the Universidad Nacional Autónoma de México. They were suspected to be hybrids between *E. isabella* and *E. lineata*, but the pale markings and other details of the forewing, the presence on the hindwing of dark rays mainly on the veins (rather than intervenally), and the form of the hindwing margin all suggest *E. vibilia* more strongly. Hybrids are clearly intermediate between the two parental species, and all are similar, suggesting that they are F_1 hybrids.

Eueides hybrid no. 6 from Costa Rica also presents difficulties. Melanic forms of *E. isabella* occur, but this is clearly different. The specimen bears a label "*Eueides isabella* x *procula*" in an unknown hand, possibly that of Keith S. Brown. The wing

shape, broad orange anal streak on the upperside forewing and the yellow costal streak on the hindwing all suggest *isabella*. The *procula* characteristics include rounded, indistinct forewing spots, hindwing yellow costal streak shortened to a pale basal spot (*procula* has a white spot here), intervenal submarginal hindwing spots on the underside that show up on the upperside through the wing tip, but not at the base, the intervenal submarginal underside hindwing spots that are rounded, and not so flat or as near the edge as in *isabella*, and the orange cell streak of the forewing underside that is mainly costal, rather than anal as in *isabella*. Finally, the male hybrid 7 from SE Brazil is clearly related to *Eueides pavana*, but has very orange forewings and lacks the prominent intervenal black rays on the hindwing. It seems fairly clear that it is a hybrid with *E. vibilia vibilia*, the males of which have orange forewing bars.

Unlike *Heliconius*, *Eueides* are drab and not favoured by collectors, and the similarity of their colour patterns may mean that hybridization in other species has been missed. Their colour patterns also make it difficult to decide which species are parents without further information. We predict that hybridization within *Eueides* is rather commoner than current records demonstrate, because of the difficulty of detecting hybrids between such similar species.

Hybrids involving "silvaniform" *Heliconius* (hybrid nos. 8-28)

Hybrids between and other *Heliconius* are readily recognized because their mimetic patterns become very disturbed. The yellow and brown ithomiine mimicry rings to which the silvaniforms belong are very different from the other crimson/orange and yellow heliconiine mimicry rings. Only two hybrids (nos. 21 & 22) have been found between any pair of ithomiine-mimicking silvaniform species, though this is likely an underestimate of the true level of hybridization between these similar appearing and closely related forms. In view of the confusion surrounding the species status of the silvaniforms, some of which are highly polymorphic, it would not be surprising if

some of the many aberrant forms among silvaniforms [1] were not produced by additional cryptic hybridization.

The remainder of the hybrids in this group are either between the red-marked silvaniform subgroup species *H. elevatus* and *H. besckei* and ithomiine-mimicking silvaniforms (nos. 14-16, 23-28), or between silvaniform species and *H. melpomene* (nos. 8-13, 17-20).

Inferences about parentage of these specimens are problematic, because the hybrids appear to be extremely rare, so that only a single specimen is known for most of the forms. For hybrid nos. 18-22 we have after careful consideration accepted the judgment of Brown [1] who reported such specimens in his review of silvaniform systematics (and to which we have added similar forms). Hybrid 11 is somewhat different from these others; it is a hybrid between a silvaniform and one of the two local rayed species, *elevatus* or *melpomene*. Its very rounded wing shape, the shape of the forewing bar, and other characteristics strongly suggest *melpomene* rather than *elevatus*, although the latter is more closely related to silvaniforms (Fig. 1; [2]). Similarly, the general darkness and the faint pale spotting in the forewing apex suggest that *H. numata aurora*, rather than another species from the area, is the silvaniform parent. Two hybrids involving a silvaniform and *H. elevatus* (nos. 14-15) have among the more dubious parentages on our list; we have seen only photos of these specimens, but we are almost certain they are hybrids. The only alternative would be that they were caused by rare mutations or variability within *H. elevatus*. If hybrids, they must represent backcrosses, because both are most similar to *H. elevatus*. Hybrid evidence for no. 15 is provided only by the a smeary red/brown forewing band in the position where *H. elevatus* is normally melanic; this is typical for certain hybrids with red-banded *H. melpomene* (for example with *ethilla* or *cydno*), but in this case it may be more likely from a more closely related silvaniform in which broken forewing bands often contain brown. Hybrid 14 is more unusual,

having very undulate distal wing margins (typical of silvaniforms such as *hecale*) as well as an extraordinary (for this area and mimetic group) broken yellow forewing band (again typical of a number of silvaniforms). Unfortunately, we know of no collections of *elevatus* or other *Heliconius* from Puerto Inírida with which to compare this solitary specimen, so it remains enigmatic. These two hybrids are included as "possibles", because it would not be at all surprising that *H. elevatus*, included within the silvaniform group (see Fig. 1), hybridizes with other members of the group. In contrast, a Bolivian specimen in the Vienna Museum (hybrid no. 16) has a much clearer status: it is labeled "*H. quitalenus sisyphus* x *H. melpomene penelope*" and has a variety of intermediate characters which point clearly to it being an unusual hybrid; Andrew Brower, who photographed this specimen, stated (*in litt.*, 1 July 2000) "I think it is pretty clearly a *hecale* x *elevatus* [hybrid]," an opinion with which we concur.

A number of hybrids in this group are found between *H. melpomene* and the silvaniforms *H. numata* or *H. ethilla* (nos. 8-11, 17-20, respectively). These extremely rare phenotypes have silvaniform patterns, and often sport unusual smeared orange forewing bands and other pattern disturbances. The orange forewing bands in most of these specimens (nos. 8, 17-20) have almost certainly been obtained from the red-banded *H. melpomene melpomene* which occurs in the areas of capture. The remaining hybrids (nos. 9-11) lack orange forewing bands, as expected, since their parents are from *melpomene* races with yellow forewing bands. Most of the specimens are probably F_1 hybrids; however, hybrid nos. 19 and 20 both look most similar to their *ethilla* parent, and we interpret them as backcrosses to *ethilla* on the basis of similar backross phenotypes found in *cydno x melpomene* crosses (below).

Finally, hybrids between *Heliconius ethilla narcaea* and *H. besckei* are known from a number of locations in S.E. Brazil (nos. 23-28). There can be no doubt from the general silvaniform pattern, coupled with the orange forewing bar and the

submarginal pale loop, characteristic of *besckei*, on the hindwing underside, that these forms are indeed interspecific hybrids between *ethilla* and *besckei*. The hybrids are of both sexes, and nos. 23-27 are relatively homogeneous, suggesting that they are all F_1 hybrids. Hybrid no. 28, however, shows a number of traits more similar to *besckei*, particularly dark forewings with very reduced orange-brown markings, and the yellow longditudinal forewing streak characteristic of *besckei*. The equivalent yellow forewing streak in *melpomene* is a recessive trait [3]; if this is also true in *besckei*, the specimen could only be produced in a backcross to *besckei*.

To date, we have no molecular evidence for interspecific hybridization within the silvaniforms or between silvaniforms and the *melpomene*/*cydno* group. However there is god laboratory evidence that crosses within the silvaniforms, and between the silvaniforms and *melpomene* group are possible. Lawrence E. Gilbert has produced unforced hybrids between *Heliconius ismenius* (a silvaniform) and other members of the silvaniform or *melpomene* groups (see [4]: Plate 1B; [5]: Table 30.1) in greenhouses at the University of Texas. In addition, Jean-Pierre Vesco (*in litt.*15 April 2001) has successfully crossed F1 hybrid males of *H. hecale* with *H. atthis* (both silvaniforms) with a strain of *H. melpomene* already containing an admixture of some genes from *cydno*, and also successfully backcrossed the male progeny to *hecale* (Table 1, Additional File 1). Thus even the most distant possible crosses in this section of the genus, between the *melpomene* and silvaniform groups, produce some viable and fertile offspring in the laboratory.

Hybrids involving *Heliconius melpomene* and *H. cydno, H. heurippa*, or *H. pachinus* (hybrids 29-100)

One of the two types of most abundant interspecific hybrids in collections are those between *Heliconius melpomene* and *H. cydno*. *Heliconius melpomene* is widespread in the neotropics, but *H. cydno* is restricted to the Eastern slopes of the Northern Andes westwards and southwards to the Pacific coastline in Ecuador, Colombia, and

Central America. Hybrids between *melpomene* and *cydno* are known from virtually wherever the two co-occur: Costa Rica, Panama, West Colombia, West Ecuador, the Magdalena Valley of Colombia, and on the eastern slopes of the Andes in Venezuela and Colombia (*melpomene* x *heurippa*). They have been collected on numerous occasions by many different people.

The key evidence for hybridization between these two species is intermediacy in pattern. Usually, they are recognized by the presence of both a white (or yellow) forewing band, as in *cydno*, and a red outer band, as in *melpomene*. There are usually many other intermediate characteristics; for example, in F_1 hybrids and some backcrosses (e.g. hybrid no. 29), the paired brown underside marks on the hindwing are often reduced as compared to *H. cydno*, where these marks are normally found, and the pale hindwing bar of some races of *cydno* is absent, or expressed as a faint "shadow" (e.g. no. 45). We also have good laboratory evidence for the genetics of hybridization in this group. Hybrids and backcrosses between *melpomene* and *cydno* have been produced in Liverpool in the 1970s (Brakefield, unpublished), in Texas [4,6-8], in Colombia [6,9], in Panama [10-12], and in France (Jean-Pierre Vesco pers. comm., Additional File 1). In the laboratory, F1 hybrids are normally produced by a female *cydno* x male *melpomene*; the reciprocal cross seems much more difficult ([11]; L. Gilbert, M. Linares, pers. obs.). Hybrids can be of either sex, but mated female hybrids typically produce no eggs [11]. Males, however, are fertile, and can be backcrossed in either direction. Thus genes (except for mitochrondrial genes, or genes on the W chromosome, because females are heterogametic) are readily transferrable from one species to another, and molecular evidence for natural hybrids in the field and introgression of some nuclear genes has now been obtained [9,13,14].

Only four *cydno x melpomene* hybrids are known from Central America (nos. 29-32). Most are apparently backcrosses to *H. cydno*, judging from the very broad, *cydno*-like pale forewing band, ringed by a narrow red outer strip (nos. 29-30, 32). This

phenotype is similar to that found in backcross hybrids between Amazonian and extra-Amazonian races of *Heliconius melpomene* [3,15]; here the colour pattern genotype is apparently homozygous for the pale-band allele N^N, i.e. $N^N N^N$ *B*-, and this is readily reproduced in laboratory hybrids [12]. In contrast, inferred F_1 hybrids (no. 31) represent $N^N N^B$ *B*- genotypes, i.e. heterozygotes for the *N* locus [12]. Similar genotypes can be produced in crosses between races of *melpomene*: heterozygotes at locus *N* have a broader red band and a more or less suppressed pale band on the forewing [3,15]. The genetics of these colour pattern differences in *H. melpomene* are therefore well understood and have been mapped in *H. melpomene*, *H. numata*, *H. cydno* and *H. pachinus* [16-19]. Central Colombian (nos. 41-65) and Venezuelan hybrids (nos. 87-96) are similar to those from Central America, but more numerous in collections. F_1, as well as backcross (to *cydno*) phenotypes appear frequently among these forms

There are also many hybrids known from western Colombia, i.e west of or in the westernmost Andes, near Cali (nos. 66-86). These hybrids are extraordinarily diverse, in part because of the huge local diversity of colour patterns in one of the parent species. *Heliconius cydno* in the Cali area has a three-way hybrid zone between a northern Cauca Valley form (*cydnides,* mimicking *H. eleuchia eleuchia*), a polymorphic southern Cauca subspecies (*weymeri* and f. *gustavi*) which mimicks the ithomiine *Elzunia humboldt* and *Heliconius erato chestertonii*, and a western form (*zelinde*) mimicking *Heliconius sapho chocoensis* and *H. eleuchia eleusinus* [20-22]. Subspecies *zelinde* meets the three Cauca forms in upper parts of the pass through the Western Cordillera formed by the Río Dagua, forming a three-way hybrid zone in *H. cydno* [6,20,23]. The Cauca Valley at this latitude lacks *H. melpomene*, but *melpomene* is found to the west of the cordillera, and meets *H. cydno* in the Dagua valley pass, thus enabling interspecific hybridization with a huge diversity of four *H. cydno* forms and their intraspecific hybrids. It may be no accident that hybrids are common from this region; if reinforcement has occurred, the presence of *cydno*

genomes from outside the area of sympatry with melpomene may allow more frequent hybridization than would be the case for forms of *cydno* that are normally sympatric with *melpomene*. Most of these specimens are clearly hybrids, and include both F_1s and backcrosses. However nos. 57, 60-62, and 64-66 75, 78-80, and 82-84 can easily be confused with pure *H. c. weymeri*. We consider them interspecific hybrids (backcrosses to *cydno*), because they display two traits that could only have come from *H. melpomene vulcanus*: a thinner yellow hindwing bar than found in *H. c. weymeri* or *H. c. gustavi* (nos. 75, 80), and heavier expression of this bar on the underside (nos. 79, 84) or, more convincingly, a combination of both traits (nos. 78, 82, 83). Both these traits are characteristic of *H. melpomene vulcanus* from W. Colombia. Laboratory crosses have reproduced these effects (M. Linares, unpub.).

Hybrids also seem relatively frequent in the lowlands and foothills of the western Andes in Ecuador (nos. 33-40), where *H. cydno alithea* is polymorphic, with a white form mimicking *H. sapho candidus* and a yellow form mimicking *H. eleuchia primularis* [24] . Some of these are clear F_1 phenotypes (nos. 34-35), but the rest are morphologically closer to *melpomene*, and we presume them to be backcrosses. None of the backcrosses to *melpomene* have the brown underside markings of *cydno*, but they have a number of other features almost certainly caused by introgression from *cydno*. These are: very strong white (or yellow) markings proximal to the red forewing band (nos. 33-37, 39, 40), yellowness of these marks (marks are always white in W. Ecuadorean *H. m. cythera*, but yellow in the yellow form of *H. cydno*) (no. 33), broadened hindwing marginal bands (no. 33), strong white or submarginal spotting on the forewing (nos. 33, 39, 40), and strong reduction of the underside yellow hindwing bar (no. 33), in some cases to a “shadow” (nos. 37, 38).

The remaining specimen in this group is no. 97. This was collected at a well-known locality for *H. heurippa* (itself a stable species probably deriving originally from a *H. cydno* x *melpomene* hybridization [9]) as well as for *H. m. melpomene*. Laboratory-

reared F_1 hybrids between these species look extremely similar to red-banded *H. melpomene melpomene* (Linares, unpub.). See also hybrid no. 92, which is between *H. cydno cordula* (similar in its yellow marks to *H. heurippa*) and *H. m. melpomene*: the similarity of no. 92, which has only a faint smear of yellow on the upperside, to pure *melpomene melpomene* shows how difficult it can be to detect this kind of hybrid. The abundance of yellow on no. 97 thus suggests that the specimen is the result of a backcross to *heurippa*. Once again, hybrids produced in the laboratory confirm the possibility of hybridization and expected phenotypes (M. Linares, unpub.).

The hybrids between the Pacific *H. pachinus* and the Atlantic *H. cydno galanthus* (98-100) were found in the region of a low pass in the mountains connecting the lowland coastal areas. The broadly blocked underside brown markings are characteristic of some hybrids between *melpomene* and *cydno* (e.g. nos. 69, 73, 81). When inbred, *Heliconius cydno* from this region produced phenotypes never before seen in the wild, having a broad looping white hindwing bar in the full region of the hindwing normally occupied by the underside brown markings, on both upper and underside (not illustrated – L. Gilbert, pers. comm.). These phenotypes (no. 98-99) are interpreted as due to expression of the submarginal pale bar from *pachinus* on a *cydno* genetic background, and can be reproduced in laboratory crosses [8].

Natural hybridization within the *melpomene*/*cydno* group can rarely be confirmed using molecular markers, although good molecular evidence for introgression between the species now exists in Costa Rica and Panama [13,14]. The rarity of natural hybrids normally makes collecting fresh hybrid material difficult. However, in one unusual site at San Cristobal, Venezuela, hybrids form about 8% of the population ([9]; Additional File 1). Microsatellite markers suggest that most of the hybrid phenotypes are late-generation backcrosses, but the normally distinct mtDNA markers are found in the "wrong" species, at this site only [9].

Although *Heliconius cydno* x *H. melpomene* hybrids are the commonest of any pair of species, they are still normally extremely rare. If we exclude the San Cristobal site mentioned above, in spite of our having collected, marked and recaptured many thousands of individual *Heliconius*, we have not ourselves found any natural F_1 hybrids between the two species. In published population studies, Smiley (1978) marked 135 *cydno* and 61 *melpomene* in the OTS field station of La Selva, on the Atlantic slopes of Costa Rica. Work in the late 1970s and early 1980s in the Osa Peninsula led to marking of 873 *H.* (*cydno*) *pachinus* and 249 *H. melpomene* (Gilbert, 1991). Kapan's study of *H. cydno* polymorphism in W. Ecuador [24] led to the marking of 2513 *cydno* and 90 *melpomene*, as well as two backcross hybrids most easily confusable with *melpomene* (nos. 39-40). Overall, these field studies have therefore scanned 3521 *cydno*/*pachinus* and 400 *melpomene* from zones of sympatry, and only two hybrids have resulted. The fraction of hybrids in natural sympatric populations is therefore of the order of 0.05%. Because Kapan [24] concentrated his efforts in habitats more suitable for *H. cydno*, it is probable that the frequency of hybrids may be somewhat higher in areas of 50:50 overlap.

Hybrids among species in the *Heliconius erato* group (hybrid nos. 101-157)

Hybrids between *H. erato* and *H. himera* have been extensively studied [5,25-30]. *Heliconius himera* is the sister taxon to *H. erato*, and its distribution in dry forest of the Huancabamba depression of northern Ecuador and southern Peru abuts with that of *H. erato*, which occurs in Ecuador both west and east of the Andes, and also east of the Andes in Peru. 57 hybrids are known from all three contact zones: in W. Ecuador with *H. erato cyrbia*, in the lower Marañon of E. Peru with *H. e. lativitta*, and in the upper Río Mayo drainage with *H. e. favorinus*. In spite of the existence of these parapatric zones of contact, comparable to the hybrid zones between geographic races of *H. erato*, we regard *H. himera* operationally as a separate species because hybrids

are always rare compared with the parental forms, unlike in the more classic hybrid zones where *H. erato* subspecies meet [5].

The best studied hybrid zone is that in W. Ecuador with *H. e. cyrbia*. We know of 52 hybrid specimens from this area (Table 1, nos. 101-152; see also [25,27,30]). Hybrids in the centre of the narrow hybrid zone make up ~10% of the combined population of the two species, about evenly split between F_1s and backcross phenotypes, the remaining 90% being parental [30]. Mitochondrial DNA and allozyme differences are retained in most "pure" individuals from the hybrid zone: the lack of morphological intergradation is paralleled by only occasional introgression throughout large portions of the genome [28]. Crosses in the laboratory occur in both directions, and the viability and fertility of hybrids is indistinguishable from that of parental species [29]. Natural hybrids are readily recreated in the laboratory, and the genetics of the colour pattern differences between the two species is known and has been mapped [31-33]. Instead, the absence of a randomly-mating hybrid swarm in the centre of the hybrid zone must be due to two major groups of factors. Firstly, strong mate choice expressed in both laboratory [29] and field [30] ensures that mating is 92-95% assortative. Secondly, there is as yet unidentified ecological selection against hybrids, presumably a mixture of predation on rare hybrids with poor warning signals, and environmental selection due to temperature and humidity [29,30,34].

The Peruvian hybrid zones between *H. himera* and the other two races of *erato* are much less well studied: only 5 hybrids are known (nos. 153-157). However both zones have been visited by JM. Hybridization between *H. himera* and *H. e. favorinus* occurs in wet habitat at about 1600m near Rodriguez de Mendoza (6°24' S, 77°29' W) and Omia in the Río Huambo valley (whose waters eventually flow into the Río Huallaga via the Río Huallabamba). Rodriguez de Mendoza is accessible via a low mountain pass into the drier Río Marañon drainage near Chachapoyas, Amazonas (6°14' S, 77°52' W) where *H. himera* is abundant. *Heliconius himera* does not occur in

the Rodriguez de Mendoza area, but individuals must occasionally fly over the 2100m pass (approx. 6°24' S, 77°37' W) into the Río Huambo valley, where *H. e. favorinus* is common. Four hybrids are known, of which one (no. 153) is almost certainly an F_1; the rest are backcrosses to *H. erato* (nos. 154-156); this is the expected direction of backcross given that no parental *H. himera* have been found in this area. The frequency of hybrids again appears low; J.M. found only one hybrid (caught by a small boy in a hat) along with 10 *H. erato favorinus* from the region. In contrast, only one male hybrid (no. 157) is known from where the Río Marañon valley emerges from the Andes, downriver from Bagua, Amazonas. This hybrid is, judging from existing laboratory crosses with *erato* and *himera* [31,33], an F_1. Hybrid frequencies must again be low: a collection in 1984-1986 of 7 *erato lativitta* and 77 *himera* from within 20 km of the overlap revealed no hybrids, even though individuals of parental species were found at the same sites [26].

Only one putative hybrid, presumably an F_1, is known between *H. erato* and *H. charithonia*, from southern Mexico (no. 158). This form was previously recorded as an aberration of *H. erato* [35]. The expression of the narrow split yellow forewing band shape is more prominent on the underside than on the upperside; this feature is also true of heterozygotes of the gene *Sd* in many interracial crosses between Amazonian yellow-banded and extra-Amazonian red-banded *H. erato.* In interracial crosses, the *Sd* gene also reduces the expression of the yellow hindwing bar of *erato* [3,15], again as seen here. However, no geographic races of *H. erato* exist with yellow band shapes approaching those of hybrid no. 158 anywhere near Mexico; the nearest yellow-banded *erato* populations are in the Amazon basin. The shape of the band on the underside clearly indicates that it must come from *charithonia*. Both *erato* and *charithonia* are extremely abundant in Mexico. Two other features that suggest *charithonia* as the other parent are the undulate hindwing margin and the generally elongated wing shape.

Evidence for hybridization between *H. charithonia* and *H. peruvianus* (159) is still scanty [36]. These two species were until recently recognized as geographic races of a single species, *H. charithonia*. However, *H. charithonia* is quite homogeneous both in allozymes and mtDNA sequence throughout its range from N. Peru to Central America, Florida and the Caribbean; and strongly divergent from *H. peruvianus* [36]. The two forms are predominantly parapatric: *H. peruvianus* is a Müllerian mimic of *Elzunia pavonii* and is restricted with its co-mimic to drier parts of W. Ecuador, S. Ecuador and N. Peru, while *H. charithonia* is found in wetter parts of the same region, and is widespread in N.E. Ecuador, N. into Central America, as well as on the Eastern slopes of the Andes from N. Peru Northwards. Thus *H. peruvianus* is closely analogous in its restricted dry forest distribution, with respect to *H. charithonia*, as *H. himera* is to *H. erato*. However, there are differences, and *H. peruvianus* is sympatric with *H. erato* in many areas of W. Ecuador, while *H. charithonia* overlaps with *H. himera* in N.E. Peru. We regard *H. peruvianus* operationally as a separate species from *charithonia* because of overlap or near-overlap with little hybridization, as in *himera* versus *erato*. Hybrids between this pair of species are unusual: no museum specimens of morphological intermediates are known, nor are there any laboratory studies confirming their possibility. The only evidence to date is from allozyme data on a single individual in an area of overlap [36]; since the hybrid appears phenotypically identical to *H. charithonia*, this individual must be the progeny of a backcross.

The final hybrids (nos. 160-161) are between *H. hecalesia* and a pair of geographic replacement forms: *H. hortense* from Mexico and Guatemala, and *H. clysonymus* from Costa Rica south to Peru. *Heliconius hortense* and *H. clysonymus* are normally recognized as separate species, and differ primarily in wing shape, although they have similar colour pattern and ecology. Both species are most abundant at 800m-1500m above sea level, and *H. clysonymus* is not known to be in contact with *H. hortense* across the lowland plains of central Nicaragua [37]. Molecular studies (Fig. 1) and the

presumably homologous colour patterns between the two species suggest that the two fall within the *H. erato* group, including *H. telesiphe* and *H. hecalesia*. Because *hecalesia* mimics *Tithorea* and other ithomiine models, while *hortense/clysonymus* have their own striking red-and-black, non-mimetic, but purely heliconiine colour pattern, specimens 160 and 161 are unmistakably hybrids of known parentage, even though supporting laboratory data does not yet exist.

References

1. Brown KS: **An illustrated key to the silvaniform *Heliconius* (Lepidoptera: Nymphalidae) with descriptions of new subspecies.** *Trans Amer Entomol Soc* 1976, **102:** 373-484.

2. Beltrán M, Jiggins CD, Brower AVZ, Bermingham E, Mallet J: **Do pollen feeding and pupal-mating have a single origin in *Heliconius*? Inferences from multilocus sequence data.** *Biol J Linn Soc* 2007, **xxx:** 000.

3. Sheppard PM, Turner JRG, Brown KS, Benson WW, Singer MC: **Genetics and the evolution of muellerian mimicry in *Heliconius* butterflies.** *Philosophical Transactions of the Royal Society of London Series B- Biological Sciences* 1985, **308:** 433-613.

4. Gilbert LE: **The biology of butterfly communities.** In *The Biology of Butterflies*. Edited by Vane-Wright RI. London: Academic Press; 1984:41-54.

5. Mallet J, McMillan WO, Jiggins CD: **Mimicry and warning color at the boundary between races and species.** In *Endless Forms: Species and Speciation*. Edited by Howard DJ. New York: Oxford Univ. Press; 1998:390-403.

6. Linares M: *Adaptive microevolution through hybridization and biotic destruction in the neotropics.* University of Texas at Austin; 1989. Ph.D.

7. Nijhout HF, Wray GA, Gilbert LE: **An analysis of the phenotypic effects of certain colour pattern genes in *Heliconius* (Lepidoptera: Nymphalidae).** *Biol J Linn Soc* 1990, **40:** 357-372.

8. Gilbert LE: **Adaptive novelty through introgression in *Heliconius* wing patterns: evidence for a shared genetic "toolbox" from synthetic hybrid zones and a theory of diversification.** In *Ecology and Evolution Taking Flight: Butterflies as Model Systems*. Edited by Boggs CL. Chicago: University of Chicago Press; 2003:281-318.

9. Mavárez J, Salazar C, Bermingham E, Salcedo C, Jiggins CD, Linares M: **Speciation by hybridization in *Heliconius* butterflies.** *Nature* 2006, **441:** 868-871.

10. Naisbit RE, Jiggins CD, Mallet J: **Disruptive sexual selection against hybrids contributes to speciation between *Heliconius cydno* and *H. melpomene*.** *Proc Roy Soc Lond B* 2001, **268:** 1849-1854.

11. Naisbit RE, Jiggins CD, Linares M, Mallet J: **Hybrid sterility, Haldane's rule, and speciation *Heliconius cydno* and *H. melpomene*.** *Genetics* 2002, **161:** 1517-1526.

12. Naisbit RE, Jiggins CD, Mallet J: **Mimicry: developmental genes that contribute to speciation.** *Evol Devel* 2003, **5:** 269-280.

13. Bull V, Beltrán M, Jiggins CD, McMillan WO, Bermingham E, Mallet J: **Polyphyly and gene flow between non-sibling *Heliconius* species.** *BMC Biol* 2006, **4:** 11.

14. Kronforst MR, Young LG, Blume LM, Gilbert LE: **Multilocus analysis of admixture and introgression among hybridizing *Heliconius* butterflies.** *Evolution* 2006, **60:** 1254-1268.

15. Mallet J: **The genetics of warning colour in Peruvian hybrid zones of *Heliconius erato* and *H. melpomene*.** *Proc Roy Soc Lond B* 1989, **236:** 163-185.

16. Jiggins CD, Mavárez J, Beltrán M, McMillan WO, Johnston JS, Bermingham E: **A genetic linkage map of the mimetic butterfly, *Heliconius melpomene*.** *Genetics* 2005, **171:** 557-570.

17. Joron M, Papa R, Beltrán M, Chamberlain N, Mavárez J, Baxter S *et al.*: **A conserved supergene locus controls colour pattern diversity in *Heliconius* butterflies.** *Public Library of Science - Biology* 2006, **4:** e303.

18. Kronforst MR, Young LG, Kapan DD, McNeely C, O'Neill RJ, Gilbert LE: **Linkage of butterfly mate preference and wing color preference cue at the genomic location of *wingless*.** *Proc Natl Acad Sci , USA* 2006, **103:** 6575-6580.

19. Kronforst MR, Kapan DD, Gilbert LE: **Parallel genetic architecture of parallel adaptive radiations in mimetic *Heliconius* butterflies.** *Genetics* 2006, **174:** 535-539.

20. Linares M: **Origin of neotropical mimetic diversity from a three-way hybrid zone of *Heliconius cydno* butterflies.** In *Tropical Diversity and Systematics*. Edited by Ulrich H. Bonn: Zoologisches Forschunginstitut und Museum Alexander Koenig; 1997:93-108.

21. Linares M: **The ghost of mimicry past: laboratory reconstitution of an extinct butterfly 'race'.** *Heredity* 1997, **78:** 628-635.

22. Merchán HA, Jiggins CD, Linares M: **A narrow *Heliconius cydno* (Nymphalidae; Heliconiini) hybrid zone with differences in morph sex ratios.** *Biotropica* 2004, **37:** 119-128.

23. Constantino LM, Salazar JA: **Natural hybridization of *Heliconius cydno* Doubleday from Western Colombia (Lepidoptera: Nymphalidae: Heliconiinae).** *Bol Cient Mus Hist Nat Univ Caldas* 1998, **2:** 41-45.

24. Kapan DD: **Three-butterfly system provides a field test of Müllerian mimicry.** *Nature* 2001, **409:** 338-340.

25. Descimon H, Mast de Maeght J: **Semispecies relationships between *Heliconius erato cyrbia* Godt. and *H. himera* Hew. in southwestern Ecuador.** *J Res Lepid* 1984, **22:** 229-239.

26. Mallet J: **Speciation, raciation, and color pattern evolution in *Heliconius* butterflies: evidence from hybrid zones.** In *Hybrid Zones and the Evolutionary Process*. Edited by Harrison RG. New York: Oxford University Press; 1993:226-260.

27. Jiggins CD, McMillan WO, Neukirchen W, Mallet J: **What can hybrid zones tell us about speciation? The case of *Heliconius erato* and *H. himera* (Lepidoptera: Nymphalidae).** *Biol J Linn Soc* 1996, **59:** 221-242.

28. Jiggins CD, McMillan WO, King P, Mallet J: **The maintenance of species differences across a *Heliconius* hybrid zone.** *Heredity* 1997, **79:** 495-505.

29. McMillan WO, Jiggins CD, Mallet J: **What initiates speciation in passion-vine butterflies?** *Proc Natl Acad Sci , USA* 1997, **94:** 8628-8633.

30. Mallet J, McMillan WO, Jiggins CD: **Estimating the mating behavior of a pair of hybridizing *Heliconius* species in the wild.** *Evolution* 1998, **52:** 503-510.

31. Jiggins CD, McMillan WO: **The genetic basis of an adaptive radiation: warning colour in two *Heliconius* species.** *Proc Roy Soc Lond B* 1997, **264:** 1167-1175.

32. Tobler A, Kapan DD, Flanagan NS, Gonzalez C, Peterson E, Jiggins CD *et al.*: **First-generation linkage map of the warningly colored butterfly *Heliconius erato*.** *Heredity* 2005, **94:** 408-417.

33. Kapan DD, Flanagan NS, Tobler A, Papa R, Reed RD, cevedo Gonzalez J *et al.*: **Localization of Müllerian mimicry genes on a dense linkage map of *Heliconius erato*.** *Genetics* 2006, **173:** 735-757.

34. Davison A, McMillan WO, Griffin AS, Jiggins CD, Mallet JLB: **Behavioural and physiological adaptation between two parapatric *Heliconius* species (Lepidoptera: Nymphalidae).** *Biotropica* 1999, **31:** 661-668.

35. de la Maza R: *Mariposas Mexicanas*. México, D.F.: Fondo de Cultura Económica S.A. de C.V.; 1991.

36. Jiggins CD, Davies N: **Genetic evidence for a sibling species of *Heliconius charithonia* (Lepidoptera: Nymphalidae).** *Biol J Linn Soc* 1998, **64:** 57-67.

37. Brown KS: *Ecologia Geográfica e Evolução nas Florestas Neotropicais*. Campinas, Brazil: Universidade Estadual de Campinas; 1979.
