# Supplementary material for: Natural hybridization in heliconiine butterflies: the species boundary as a continuum
Source: BMC Evol Biol. 2007 Feb 23;7:28. doi: 10.1186/1471-2148-7-28 (PMC1821009; doi:10.1186/1471-2148-7-28)
Supplement: Additional File 3 — Distance measures for mtDNA among species of Heliconiina. PDF document containing table with details of average raw % DNA divergence, based on 1569 bp of the genes CoI, tRNA-leu, and CoII. [file 1471-2148-7-28-S3.pdf]

Natural hybridization in heliconiine butterflies: the species boundary as a continuum

by James Mallet, Margarita Beltrán, Walter Neukirchen, and Mauricio Linares

Additional file 3 – Distance measures for mtDNA among species of Heliconiina

The figures given are for average uncorrected (raw) % divergence between pairs of species which overlap, or average divergences among individuals of the same species sampled. Based on 1569 bp of CoI/CoII mtDNA sequences (Beltran et al. 2006).

A. *Heliconius erato-sara* group

	ant	hew	con	eleu	sap	sar	leu	dem	ric	cha	per	era	him	cly	hor	hecs	tel
antiochus	*1.35*																
hewitsoni	x	*0.00*															
congener	x	x	x														
eleuchia	x	x	x	*0.00*													
sapho	6.77	x	x	7.73	*0.32*												
sara	5.90	5.77	7.19	7.48	6.80	*0.78*											
leucadia	7.35	x	x	x	x	5.87	x										
demeter	8.43	x	x	x	x	8.56	9.71	*0.26*									
ricini	7.80	x	x	x	x	7.96	x	x	*0.39*								
charithonia	x	x	x	x	x	7.37	x	x	x	*0.60*							
peruvianus	x	x	x	x	x	x	x	x	x	**3.76**	*0.06*						
erato	8.28	8.62	9.96	9.66	9.07	8.26	9.17	8.93	8.43	**7.58**	x	*2.30*					
himera	x	x	x	x	x	x	x	x	x	7.51	8.52	**3.20**	*0.77*				
clysonymus	x	x	x	x	x	x	x	x	x	7.12	x	7.08	x	*0.64*			
hortense	x	x	x	x	x	8.84	x	x	x	7.49	x	6.98	x	x	x		
hecalesia	x	x	x	9.90	9.19	8.38	x	x	x	7.54	x	5.74	x	**7.22**	**6.77**	*0.32*	
telesiphe	x	x	10.72	x	x	x	x	x	x	7.37	x	7.40	x	5.67	x	x	*0.26*

Heliconius

KEY	
5.28	= Av. % divergence (uncorrected)
x	= no data available, or not sympatric
1.02	= intraspecific divergence
**4.56**	= interspecific divergence, hybrids known
6.55	= interspecific divergence

B. *Heliconius melpomene-cydno*-silvaniform group

melpomene	*1.53*																			
cydno	**3.15**	*1.22*																		
pacchinus	3.27	**1.37**	*1.74*																	
heurippa	**3.17**	x	x	*0.06*																
elevatus	5.17	x	x	x	*0.19*															
atthis	5.16	5.44	x	x	x	*0.46*														
hecale	**4.93**	5.38	5.86	5.47	**1.67**	1.05	*0.06*													
pardalinus	5.10	x	x	x	1.58	x	1.64	x												
ethilla	**5.34**	5.52	x	5.80	3.32	3.19	3.16	3.09	*1.48*											
besckei	5.32	x	x	x	x	x	x	x	**3.56**	*0.26*										
ismenius	5.35	5.70	6.15	x	x	3.94	3.83	x	4.66	x	*0.32*									
numata	**5.77**	5.89	x	6.05	4.41	x	4.28	4.18	**4.52**	4.47	x	*0.39*								
metharme	8.46	x	x	x	8.21	x	7.79	7.76	8.24	x	x	8.44	*0.32*							
aoede	7.65	x	x	x	7.60	x	7.83	7.56	7.53	x	x	8.56	6.86	*0.52*						
			x	x																
burneyi	6.24	x	x	x	5.80	x	6.05	5.76	6.73	x	x	6.37	8.50	7.56	*0.26*					
wallacei	6.45	x	x	x	6.25	x	6.00	5.96	6.73	x	x	6.73	7.98	7.06	3.45	*0.19*				
egeria	7.41	x	x	x	7.71	x	7.28	7.38	7.94	x	x	7.87	8.69	8.70	5.94	5.91	x			
hecuba	7.03	7.12	x	x	x	x	x	x	x	x	x	x	x	x	x	x	x	*0.19*		
xanthocles	7.73	7.57	x	7.82	8.15	x	8.05	8.18	8.24	x	x	8.69	9.40	8.38	8.31	7.44	8.81	x	*0.26*	
doris	8.29	8.02	8.47	8.17	8.79	8.20	8.27	8.44	8.60	x	8.64	8.85	8.96	8.63	8.09	7.61	8.47	6.92	7.65	*0.64*
	mel	cyd	pac	heu	elev	att	heca	par	eth	bes	ism	num	met	aoe	bur	wal	ege	hecu	xan	dor
	Heliconius												Neruda		Heliconius					Laparus

C. *Eueides* species

procula	*0.84*							
vibilia	4.99	*0.32*						
pavana	x	**2.87**	x					
isabella	**4.19**	**4.09**	3.73	*0.13*				
lineata	5.35	6.73	x	6.12	x			
aliphera	5.54	6.47	6.09	5.80	6.86	*0.13*		
lybia	6.76	**7.15**	6.70	6.47	7.44	5.94	*1.93*	
tales	x	8.26	x	7.90	x	6.77	7.25	*2.17*
	proc	vib	pav	isa	lin	ali	lyb	tal
	Eueides							
